# Biosynthesis and characterization of silver nanoparticles synthesized using extracts of *Agrimonia eupatoria* L. and *in vitro* and *in vivo* studies of potential medicinal applications†

**DOI:** 10.1039/d3ra07819a

**Published:** 2024-02-05

**Authors:** Katarina Marković, Ana Kesić, Mirjana Novaković, Mirjana Grujović, Dušica Simijonović, Edina H. Avdović, Sanja Matić, Milica Paunović, Milena Milutinović, Danijela Nikodijević, Olgica Stefanović, Zoran Marković

**Affiliations:** a University of Kragujevac, Institute for Information Technologies, Department of Science Jovana Cvijica bb 34000 Kragujevac Serbia akesic@uni.kg.ac.rs; b University of Belgrade, Vinca Institute of Nuclear Sciences – National Institute of the Republic of Serbia, Department of Atomic Physics Belgrade Serbia; c University of Kragujevac, Faculty of Science, Department of Biology and Ecology Radoja Damjanovic 12 Kragujevac Serbia

## Abstract

This research explores the synthesis, characterization, and biological activities of silver nanoparticles (AgNPs) derived from acetone (AgNPs-acetone) and aqueous (AgNPs-H_2_O) extracts of *Agrimonia eupatoria*. The nanoparticles exhibit isometric morphology and uniform size distribution, as elucidated through Transmission Electron Microscopy (TEM) and high-resolution TEM (HRTEM) analyses. The utilization of Scanning Transmission Microscopy (STEM) with High-Angle Annular Dark-Field (HAADF) imaging and energy dispersive spectrometry (EDS) confirms the crystalline nature of AgNPs. Fourier Transform Infrared (FTIR) analysis reveals identical functional groups in the plant extracts and their corresponding AgNPs, suggesting the involvement of phytochemicals in the reduction of silver ions. Spectrophotometric monitoring of the synthesis process, influenced by various parameters, provides insights into the kinetics and optimal conditions for AgNP formation. The antioxidant activities of the plant extracts and synthesized AgNPs are evaluated through DPPH and ABTS methods, highlighting AgNPs-acetone as a potent antioxidant. Third-instar larvae exposed to the extracts have differential effects on DNA damage, with the acetone extract demonstrating antigenotoxic properties. Similarly, biosynthesized AgNPs-acetone displays antigenotoxic effects against EMS-induced DNA damage. The genotoxic effect of water extract and AgNPs-acetone was dose-dependent. Hemolytic potential is assessed on rat erythrocytes, revealing that low concentrations of AgNPs-acetone and AgNPs-H_2_O had a nontoxic effect on erythrocytes. Cytotoxicity assays demonstrate time-dependent and dose-dependent effects, with AgNPs-acetone exhibiting superior cytotoxicity. Proapoptotic activity is confirmed through apoptosis induction, emphasizing the potential therapeutic applications of AgNPs. The antimicrobial activity of AgNPs reveals concentration-dependent effects. AgNPs-H_2_O display better antibacterial activity, while antifungal activities are comparable between the two nanoparticle types.

## Abstract 2

Silver nanoparticles were biosynthesized using extracts from the plant *Agrimonia eupatoria*. After the characterization, their antioxidant activity, antimicrobial activity, genotoxicity, cytotoxicity, and hemolytic activity were investigated.

## Graphical abstract

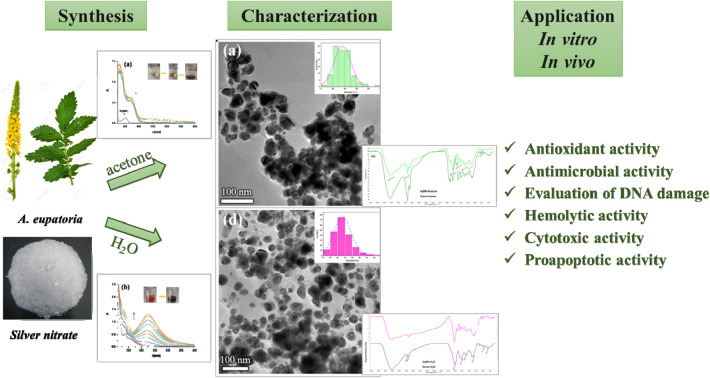

## 1. Introduction

Nanoparticles are materials that have nanoscale dimensions, with size ranging from 1 to 100 nm, so they possess exceptional physical and chemical properties, large surface area, high energy, and quantum confinement.^1^ Besides, they exhibit a variety of chemical characteristics and can be purely metallic, MtNPs, (silver, gold, copper, zinc, *etc.*), metallic oxide-based (ZnO, MgO, MnO, NiO…), silicate-based, polymeric, or carbon-based.^2^ Oxide-based nanoparticles have been extensively investigated in recent times, exhibiting a broad spectrum of applications in catalysis and environmental protection, and promising possibilities in the field of medicine.^3–6^ Many morphologies of nanoparticles, such as spheres, cylinders, sheets, or tubes, can be created.^2^ One of two strategies, a top-down approach or bottom-up strategy is used in all physical, chemical, and biological ways of synthesizing nanoparticles. In a top-down approach, the required bulk material is reduced to small particles by applying suitable lithographic methods, such as grinding and sputtering. On the other side, for bottom-up types of synthesis various chemical and biological techniques are employed, where the nanoparticles are formed by self-assembling of atoms into new nuclei, which then grow into a particle with nanoscopic dimensions.^7^

In the beginning, different techniques used for the synthesis of metal nanoparticles (MtNPs) were, in fact, only physical or chemical methods. These methods, however, have many disadvantages, including high energy consumption, the use of expensive equipment, and the use of toxic chemicals, which are hazardous to the environment.^8–10^ As an environmentally friendly alternative to MtNPs production, green synthesis methods based on the use of plants, microorganisms, enzymes, polysaccharides, and biodegradable polymers have been developed,^10,11^ where plant and animal extracts were used as reducing agents and stabilizers. Indeed, various methods for the synthesis of MtNPs were developed, due to their innovative and diverse applications in medicine, environmental science, and agriculture.^8,12^ Silver and gold nanoparticles have found extensive application in recent years for developing sensors aimed at detecting diverse toxins in water and food. This utilization is driven by the imperative to safeguard the environment amidst the prevalent overuse of pesticides and plastics.^13–16^

*Agrimonia eupatoria* L. (Rosaceae family) also known as “*agrimony*”, is utilized as a raw material for the extraction of pharmaceutical components or the manufacture of medications in the pharmaceutical industry. The plant also shows anti-inflammatory, neuroprotective, antidiabetic, hepatoprotective, and anticancer activities in addition to antioxidant and antibacterial abilities.^17^ Since, it is very interesting to examine all its application possibilities, as a reducing agent for the synthesis of various metallic nanoparticles. In the past, silver nanoparticles (AgNPs) were synthesized using a similar approach with a plant from the Rosaceae family, specifically *Rosa brunonii*, revealing notable antimicrobial properties.^18^

Most commonly, silver nanoparticles (AgNPs) are prepared by chemical reduction in organic solvents or water, resulting in a stable, colloidal dispersion. A silver metal ion solution and a reducing biological agent are the two main components needed for the green production of silver nanoparticles.^19^ Reducing agents could be found in biological systems and are discovered in four of the five types of living things: fungi, plants, unicellular organisms with genuine nuclei, and prokaryotes without true nuclei.^20^ Silver NPs are synthesized using plant extracts as a reducing agent and stabilizer (to prevent unwanted colloid agglomeration). Steroids, saponins, carbohydrates, flavonoids, and phytoconstituents, which are found in relatively high concentrations in plants, act as reducing and capping agents and thus maintain the stability of Ag nanoparticles.^21^ The synthesis and stabilization of NPs produced by biological entities are influenced by several variables, including pH, temperature, and reaction time.^22,23^ The obtained nanoparticles show significant catalytic and antibacterial activity, as well as a good potential for nanobiotechnology and medical applications.^24,25^ Due to their physical, chemical, and biological properties, Ag NPs are suitable for further research.

The aim of this paper was to examine the preparation of silver nanoparticles using the aqueous and acetone extracts of the plant *A. eupatoria*, and our preceding works contain in-depth analyses of the conditions for these synthesizers.^26,27^ The obtained nanoparticles, from acetone (AgNPs-acetone) and aqueous (AgNPs-H_2_O) extracts, underwent thorough characterization and comparison. Then, different *in vivo* and *in vitro* biological measurements were carried out. The antioxidant activity, genotoxicity, cytotoxicity, and effect of the obtained nanoparticles on erythrocytes were examined. In addition to *in vivo* research, *iv vitro* antimicrobial testing of nanoparticles was also performed. Using these methods, the overall biological potential of the obtained nanoparticles was determined.

## 2. Experimental

### 2.1. Chemicals

Silver nitrate and acetone were purchased from Sigma Aldrich, USA. Dulbecco's Modified Eagle Medium (DMEM) was obtained from Capricorn Scientific, Germany. Ethidium bromide (EB), 3-[4,5-dimethylthiazol-2-yl]-2,5-diphenyltetrazolium bromide (MTT), were obtained from SERVA, Germany. Acridine Orange (AO) was obtained from Acros Organics, New Jersey, USA. Phosphate-buffered saline (PBS) without calcium and magnesium, normal melting point agarose (NMA), low melting point agarose (LMA), and collagenase were obtained from Alfa Trade Enterprise D.O.O.; ethyl methanesulphonate (EMS) were from Sigma-Aldrich, St. Louis, MO, USA. Resazurin was obtained from Alfa Aesar GmbH & Co., Karlsruhe, Germany. The chemicals used in this study for DPPH and ABTS radical scavenging assay: 2,2-diphenyl-1-picrylhydrazyl 1,1-diphenyl-2-picrylhydrazyl radical (DPPH), 2,2′-azino-bis(3-ethylbenzothiazoline-6-sulfonic acid) diammonium salt (ABTS), referent phenolic standards (nordihydroguaiaretic acid (NDGA), (*S*)-6-methoxy-2,5,7,8-tetramethylchromane-2-carboxylic acid (Trolox)), and methanol were procured from Sigma-Aldrich Chemicals. Tetracycline, ampicillin, and amphotericin B were obtained from Sigma Chemicals Co., USA, and itraconazole from Pfizer Inc., USA. All other solvents and chemicals were of analytical grade. In distilled water, all solutions were made.

### 2.2. Preparation of plant extracts

A previously described method was used to prepare the plant aqueous and acetone extracts.^17^ The maceration process was used to extract dried, crushed plant material in acetone or distilled water. In a nutshell, 800 mL of solvent was used to soak 60 g of plant material, and the plant material was macerated three times at room temperature. A new solvent was used every 24 h. The samples were run through filter paper every 24 h and the filtrates were then collected and dried using a rotary evaporator (DLAB, RE 100 S) at 40 °C. Dry water and acetone extracts were kept at 4 °C in a refrigerator.

### 2.3. Synthesis of silver nanoparticles and optimization conditions

Silver nitrate (AgNO_3_) was used for the synthesis of Ag nanoparticles. Aqueous or acetone extracts of the plant were utilized to reduce this salt and stabilize the resulting silver nanoparticles. The procedures were carried out under strict control by a magnetic stirrer (MAGE 12/17). The optimized conditions for the synthesis were obtained by varying parameters, such as the concentration of starting components, pH value, and temperature. In both cases of synthesis, with aqueous and acetone extracts of the plant, silver nitrate was dissolved in concentrations of 5 mM, 10 mM, and 20 mM. Using a solution of 0.1 M NaOH and 0.1 M HNO_3_, the pH of the reaction mixtures was adjusted to 4, 6, and 8 for the case of aqueous extract and to 2, 4, and 6 for the acetone extract. On a magnetic stirrer, the reaction mixtures were heated under strict control to 25 °C and 50 °C. UV-vis spectrophotometry and visual color change (from light yellow to dark brown) were utilized as an indicator for the formation of AgNPs in order to optimize the conditions for the synthesis. The concentration of AgNO_3_ of 5 mM, reaction temperature of 25 °C, pH = 4, 1% of plant extract, and reaction duration of 3 h were found to be ideal conditions for the maximum yield of AgNPs using the acetone extract of plant.^26^ With the aqueous plant extract, the highest yield of AgNPs was obtained after 3 h of reaction time, at a temperature of 25 °C and pH = 6, with a 5 mM concentration of AgNO_3_, and 1% of plant extract.^27^ After AgNPs were synthesized, the suspension was centrifuged using a Scilogek|Laboratory microcentrifuge, model DM0412, for 20 minutes at 4500 rpm. The leftover material was centrifuged once, then resuspended in demineralized water. After that, the precipitated nanoparticles were dried in a hot air oven (40 °C) and stored at 4 °C in a refrigerator. Software called OriginPro 2019b – 64 bit was used to examine the data. A PerkinElmer Lambda 365 spectrophotometer was used to track the synthesis of AgNPs in the 200–800 nm wavelength range.

### 2.4. Characterization – transmission electron microscopy (TEM), UV-vis spectrophotometry, and FTIR spectroscopy

Microstructural analyses of silver nanoparticles were done on an FEI Talos F200X microscope with an X-FEG source and a maximum accelerating voltage of 200 keV. Conventional and high-resolution transmission electron microscopy (TEM/HRTEM), scanning transmission (STEM) studies and high-angle annular dark-field (HAADF) imaging with energy dispersive spectrometry (EDS) analyses for spatially resolved elemental mapping were carried out. The particle size distributions and interplanar spacing of the characteristic crystalline planes of individual nanoparticles were determined by using the ImageJ software package.^28^ For TEM observations, the samples were prepared by a standard procedure where the solid powder was first dispersed into ethanol and then a drop of the solution was applied onto a carbon-coated copper grid, which was dried in air. A PerkinElmer Lambda 365 spectrophotometer was used to track the synthesis of AgNPs in the 200–800 nm wavelength range. Obtained nanoparticles samples, as well as dry extract used in their synthesis, were subjected to Fourier transform infrared spectroscopy (FTIR, PerkinElmer Spectrum One FT-IR) for the detection of functional groups of molecules present in a sample.

### 2.5. Antioxidant activity

The antioxidant activity of obtained extracts and the appropriate nanoparticles was determined by DPPH and ABTS radical scavenging assays.^29,30^ The UV-vis measurements were recorded on PerkinElmer, Lambda 365 UV/vis Spectrophotometer.

#### 2.5.1 DPPH radical scavenging assay

The tested samples (20 μL of different concentrations dissolved in DMSO and 980 μL of methanol) were mixed with the same volume of the solution of DPPH in methanol (0.05 mM). The prepared samples were shaken well and left at room temperature in the dark for 20 min. After the incubation period, absorbance was determined at 517 nm by using the methanol as a blank control. All tests were run in triplicate and averaged. The results are presented as mean ± SD. Nordihydroguaiaretic acid (NDGA) was used as the positive control.

#### 2.5.2 ABTS radical scavenging assay

In this assay, stock solutions of ABTS (7 mM) and potassium persulfate (2.45 mM) were first prepared. The working solution was then prepared by mixing the two stock solutions in equal quantities and allowing them to react for 12–16 h at room temperature in the dark. The produced BTS radical cation (ABTS^+^˙) is diluted with methanol to obtain a solution with an absorbance of 0.70 units at 734 nm. Different concentrations of samples were prepared in DMSO. Next, 20 μL of sample and 980 μL of methanol were mixed, then an equal amount of ABTS was added and the absorbance value was measured at 734 nm.

### 2.6. Drosophila strain and evaluation of DNA damage

The fly adults and larvae of wild-type Canton-S strain of *D. melanogaster* (Bloomington Stock Centre, Indiana, USA) was raised on standard food media containing agar, sugar, and yeast in glass bottles at 25 °C, 60% humidity, and light/dark daily cycle of 12:12 h.

Third instar larvae of *D. melanogaster* (74 ± 2 h) were placed in standard *Drosophila* corn medium without or with ethyl methanesulphonate (EMS, 1 mM in PBS) and these groups were represented by negative and positive controls.^31^ Besides negative and positive controls, silver nitrate was used to determine the effects of the ionic *versus* nanoparticulated forms.^32^ For the evaluation of genotoxicity, larvae were placed separately in a medium with different concentrations of *A. eupatoria* water and acetone extracts or AgNPs from extracts (0.5, 1, and 2 mg mL^−1^ standard *Drosophila* food) and fed for 24 h. To facilitate comparisons, extracts, and AgNPs in the antigenotoxic study, at the same concentrations as for genotoxicity, were administered simultaneously with 1 mM EMS for 24 h.

After the exposure period, both control and treated larvae from each group (98 ± 2 h old) were removed from the media and the cell suspension from the anterior midguts was prepared according to Howell and Taylor^33^ modified by Mukhopadhyay *et al.*^34^ and Siddique *et al.*^35^

The alkaline comet assay was performed according to Singh *et al.*^36^ with minor modifications.^34^ The images were visualized and captured with 40× objective lens of fluorescence microscope Nikon (Ti-Eclipse) attached to CCD camera. One hundred randomly selected cells (50 cells per two replicate slides) were analyzed per treatment by a visual scoring method.^37^ The total comet score and percentage reduction (% *R*) in the total comet score was calculated by Manoharan and Banerjee^38^ and Waters *et al.*^39^

### 2.7. Estimation of hemolytic activity

All the experimental procedures involving animals were conducted in accordance with EU Directive 2010/63/EU for animal experiments and were approved by The University Ethics Committee for Animal Experimentation, University of Kragujevac. For the assessment of hemolytic activity, stock solutions of investigated substances were obtained by dissolving in phosphate buffer solution (PBS, pH = 7.4) solution at 300 μg mL^−1^ concentration. Obtained solutions for synthesized nanoparticles and AgNO_3_ were colloidal suspensions. The blood of a male Wistar albino rat was collected in a tube with anticoagulant and centrifuged at 2500 rpm for 10 min. The plasma and leukocytes/platelets layer were discarded, while precipitated erythrocytes were resuspended in an equal volume of PBS and washed three times following the centrifugation in the same conditions. Washed cells were then diluted in PBS to obtain 5% (v/v) suspension which was immediately used for further analyses.

The hemolytic activity of investigated substances was monitored through their potential ability to induce erythrocytes lysis and the subsequent release of hemoglobin upon incubation of cells under physiological conditions.^13^ All the stock solutions of investigated substances were additionally diluted with PBS and 1 mL of obtained diluted solutions were mixed with the same volume of 5% suspension of erythrocytes to attain their final concentrations of 150, 120, 90, 60, 30, 10, and 1 μg mL^−1^. The negative control was the cells treated with PBS only, while the positive control was the cells treated with 1% sodium dodecyl sulfate (SDS). The samples were then incubated simultaneously at 37 °C for one hour in a thermo-shaker which ensures gentle shaking of the test tubes. After the incubation period, the samples were centrifuged at 1200 rpm for 10 min, and the supernatant was used for spectrophotometry analyses proceed at 540 nm against the corresponding blank sample. The percentage of hemolysis was calculated by using the following equation:
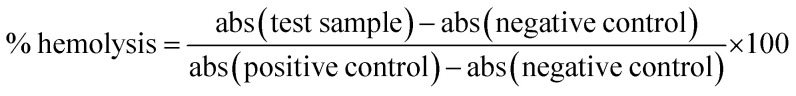


### 2.8. Determination of AgNPs effects on cancer cells

#### 2.8.1 Cell viability detection assay

The effect of silver nanoparticles, water, and acetone extract on SW-480 colorectal cancer cell viability was determined by MTT assay.^40^ After 24 h of cells seeding (in a 96-well plate, 10^4^ cells per well), they are treated by AgNPs-H_2_O and AgNPs-acetone in a concentration range of 1 to 150 μg mL^−1^. Untreated cells were used as control. The test was previously described in detail in earlier publications and was performed after 24 and 72 h.^41^ Absorbances were measured on an ELISA reader at 550 nm and results of viable cells were calculated as the ratio of observed absorbance of treated group divided by the absorbance of control group, multiplied by 100. The IC_50_ values were determined from the dose response viability curves by the CalcuSyn software.

#### 2.8.2 Analysis of cell death

The type of induced cell death was determined by the acridine orange/ethidium bromide (AO/EB) double staining assay.^42^ After 24 h of cell seeding (in a 96-well plate, 10^4^ cells per well), SW-480 cells are treated with AgNPs-H_2_O and AgNPs-acetone in an IC_50_ concentration for both extracts. Staining was performed 24 h after treatment. Untreated cells were used as controls. Immediately after staining, the cells were observed on an inverted fluorescent microscope (Nikon Ti-Eclipse), at 400× magnification. A minimum of 300 cells was counted in each sample. The number of viable cells, early and late apoptotic and necrotic cells were calculated in the percentages, concerning the total cell number per sample (minimum 300 cells).

### 2.9. Determination of antimicrobial activity

#### 2.9.1 Test microorganisms

The antimicrobial activity of nanoparticles was tested against 17 strains of bacteria and 6 strains of fungi. The list of tested microorganisms is presented in Tables 7 and 8. All clinical isolates were a generous gift from the Institute of Public Health, Kragujevac. The other microorganisms (fungi and ATCC strains) were provided from a collection held by the Microbiology Laboratory, Faculty of Science, University of Kragujevac.

#### 2.9.2 Suspension preparation

Bacterial and yeast suspensions were prepared by the direct colony method.^43^ The turbidity of initial suspensions was adjusted using a 0.5 McFarland densitometer (BioSan, Latvia). Initial bacterial suspensions contain about 10^8^ colony-forming units (CFU) per mL and yeast suspensions contain 10^6^ CFU mL^−1^ 1 : 100 dilutions of initial suspension were additionally prepared into sterile 0.85% saline. The suspensions of fungal spores were prepared by gentle stripping of spores from slopes with growing mycelia. The resulting suspensions were 1 : 1000 diluted in sterile 0.85% saline.

#### 2.9.3 Microdilution method

Antimicrobial activity was tested using the microdilution method with resazurin determining the minimum inhibitory concentration (MIC).^44^ Twofold serial dilutions of the tested nanoparticles were made in sterile 96-well microtiter plates containing 0.1 mL of Mueller–Hinton broth (Torlak, Belgrade, Serbia) per well for bacteria and 0.1 mL of Sabouraud dextrose broth (Torlak, Belgrade, Serbia) per well for fungi. The tested concentration range was from 0.078 mg mL^−1^ to 5 mg mL^−1^. The microtiter plates were inoculated with suspensions to give a final concentration of 5 × 10^5^ colony forming units (CFU) per mL for bacteria and 5 × 10^3^ CFU mL^−1^ for fungi. The growth of the bacteria and the yeasts was monitored by adding resazurin, an indicator of microbial growth. Resazurin is a blue non-fluorescent dye that becomes pink and fluorescent when reduced to resorufin by oxidoreductases within viable cells. The inoculated microtiter plates were incubated at 37 °C for 24 h for bacteria, at 28 °C for 48 h for yeasts, and at 28 °C for 72 h for fungi. MIC was defined as the lowest concentration of tested plant extracts that prevented resazurin color change from blue to pink. For fungi, MIC values of the tested plant extracts were determined as the lowest concentration that inhibited visible mycelia growth. Minimum microbicidal concentration (MMC) was determined by inoculation of nutrient agar medium by plating 10 μL of samples from wells, where no indicator color change was recorded. At the end of the incubation period the lowest concentration with no growth (no colony) was defined as minimum microbicidal concentration. Tetracycline, ampicillin, amphotericin B, and itraconazole, dissolved in the nutrient liquid medium, were used as reference compounds (concentration in μg mL^−1^). Each test included growth control and sterility control. All tests were performed in duplicate and mean values were presented.

#### 2.9.4 Statistical analysis

The results for cell viability are expressed as mean ± standard error (SE). Statistical significance was determined using the Student's *t*-test or the one-way ANOVA test for multiple comparisons. A *p* value < 0.05 was considered significant. The IC_50_ values were calculated from the dose curves by CalcuSyn. Results for evaluation of DNA damage were expressed as mean ± SEM and statistical evaluation of data was analyzed with one-way analysis (ANOVA) followed by *post hoc* multiple comparisons using SPSS statistical software package, version 13.0 for Windows. The significant differences were considered at *p* < 0.05.

## 3. Results and discussion

### 3.1. Transmission electron microscopy (TEM) characterization of AgNPs

**Fig. 1:**
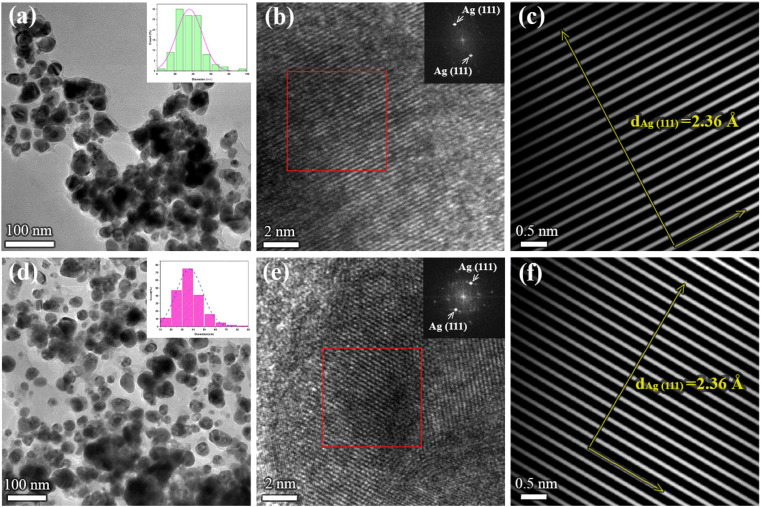
Low magnification TEM micrographs, high-resolution images with FFT patterns of the selected area of the sample (marked with rectangular in HRTEM images), and respective inverse FFT images of the Ag NPs obtained from different plant extracts. Each row corresponds to the following sample: (a–c) – Ag NPs synthesized from acetone plant extract, (d–f) – Ag NPs synthesized from aqueous plant extract (d–f). Histograms with the particle size distributions are given in the insets in (a and d).

To obtain information on the size, shape, and crystalline structure of silver nanoparticles, the samples were subjected to TEM analysis. TEM images in combination with high-resolution (HRTEM) micrographs of the samples for different plant extracts are presented in Fig. 1. Histograms with the particle size distributions are also provided and given in the same figure. For both samples, low magnification TEM micrographs (Fig. 1a and d) confirmed the formation of Ag NPs, which are characterized by high mass image contrast and observed in the images as dark features. Besides, one observes that the nanoparticles have an isometric morphology with a uniform particle size distribution. In the case of the sample synthesized from acetone plant extract (a) NPs size varies from a few nanometers up to 100 nm, the majority being with a diameter in the range of 20–50 nm. Similar results were obtained for aqueous plant extract (Fig. 1d), although in this case a larger number of nanoparticles with an average diameter of 35 ± 1 nm was formed. Similar results were given by Bhagat *et al.* (2019).^18^ The authors investigated the silver nanoparticles synthesized by chemical reaction using an aqueous extract of *Rosa brunonii* Lindl as a reducing agent. Further examinations of the samples were performed at high magnifications to ensure details on the crystalline structure of Ag nanoparticles. HRTEM image of an enlarged section of one isolated particle for the sample synthesized from acetone plant extract is given in Fig. 1b. In the image one observes clear and ordered lattice stripes, thus confirming that Ag NPs are crystalline in nature. Corresponding Fast Fourier Transform (FFT) analysis of the selected area of the image is given in the inset and presents highly bright spots which originate from (111) Ag crystal planes. Characteristic *d*-spacing was determined from respective inverse FFT image (Fig. 1c), by directly measuring the distance of 20 lattice planes, and found to be 2.36 Å at an average, agreeing with the referent value (JCPDS cards no. 04-0784). HRTEM micrograph with FFT and inverse FFT analysis of the sample for aqueous plant extract is presented in Fig. 1e and f, respectively. Again, the images revealed a highly ordered structure characterized by well-defined crystalline planes, with the measured interplanar spacing of 2.36 Å for (111) Ag.

**Fig. 2:**
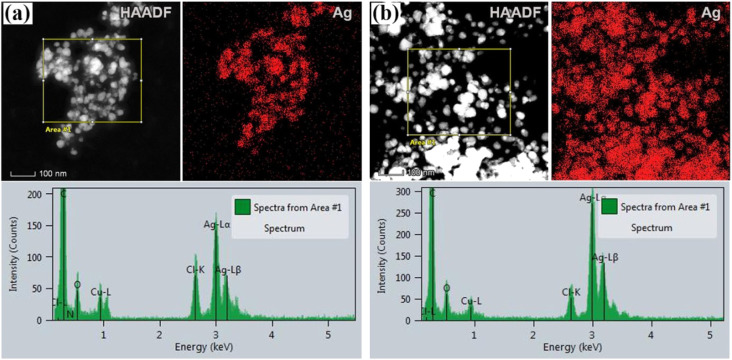
STEM analysis of silver nanoparticles. STEM/HAADF images and EDS maps are given for Ag NPs with acetone (a) and aqueous (b) plant extracts. Respective EDS spectra of the samples, shown in the bottom side of the figure, were taken from the sample area covering a large number of NPs, as indicated in HAADF images in (a and b).

Apart from the TEM examination of the samples, the chemical composition was also investigated using additional scanning transmission microscopy imaging, together with the energy dispersive spectrometry method. Fig. 2a and b present STEM/HAADF micrographs and corresponding elemental image maps obtained for NPs with acetone and aqueous plant extracts, respectively. The elemental mapping results indicated the maximum distribution of silver, suggesting that Ag was the predominant element in the respective samples. The EDS spectra, taken from the large number of particles were also measured for both plant extracts and presented in the same figure (bottom side in Fig. 2a and b). In the spectra, one observes the strong peaks at 3 keV and 3.18 keV, which originate from characteristic Lα and Lβ lines of silver. The other elements that appeared, like carbon and copper, correspond to the TEM grid used for analysis, whereas the presence of Cl might result from the incomplete reduction of different chemical compounds used during the synthesis of NPs, or could come from the matrix of the plant. Since the fraction of chlorine was determined to be approximately 7 wt% and 16 wt%, for aqueous and acetone plant extract, respectively, it is likely that in addition to Ag nanoparticles, AgCl NPs are also formed. Indeed, the formation of AgCl nanoparticles during the synthesis of Ag NPs using plants has been observed and reported earlier in the literature by other authors.^45,46^

### 3.2. FTIR analysis

**Fig. 3:**
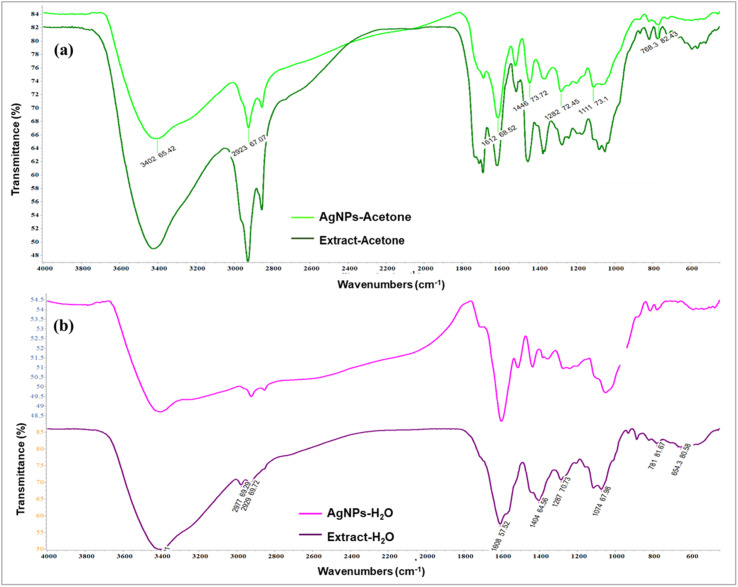
FTIR spectra of (a) acetone extract *Agrimonia eupatoria* and corresponding AgNPs (AgNPs-acetone), (b) aqueous extract *A. eupatoria* and the corresponding AgNPs (AgNPs-H_2_O).

By using FTIR analysis, it was confirmed that the biomolecules in the extracts were responsible for the reduction of silver ions and stabilization of the produced nanoparticles. It was observed that there are identical vibrational bands in the spectra of plant extracts and their corresponding nanoparticles, which means that identical functional groups from phytochemicals are present. By contrasting the detected bands with the expected values, functional groupings were found. The obtained values of the absorption bands were compared with standard values from the literature data, and the most distinctive functional groups were identified. There was a coincidence of absorption bands at 3402.65 cm^−1^ for AgNPs-acetone and 3421.03 cm^−1^ for extract-acetone, as shown in Fig. 3a. The vibration of the –OH group in phenolic compounds is caused by this band. The aliphatic –C–H stretching is represented by a band at 2926.09 cm^−1^ in the extract-acetone spectrum and 2923.67 cm^−1^ in the AgNPs-acetone spectrum, while the C–C aromatic ring is indicated by the emergence of prominent bands at 1612.66 cm^−1^ and 1615.27 cm^−1^. These broad bands are combined with the bands that originate from the stretching vibrational band at 1730 cm^−1^ which is present in both the C=O group carboxylic acid derivatives and the C=O group of the flavone ring. Bands at 1446.73 cm^−1^ and 1455.71 cm^−1^ in the AgNPs-acetone and extract-acetone spectra, respectively, could be the result of carboxylic acid or ester C–O bending vibrations and alcohol or phenol O–H bending vibrations. Similar results were observed for the aqueous extract of the plant and equivalent nanoparticles (Fig. 3b). In particular, AgNPs-H_2_O and extract-H_2_O spectra contain bands at 3401.79 cm^−1^ and 3401.01 cm^−1^, respectively, corresponding to the stretching vibrations of O–H groups from phenolic compounds (flavonoids, phenolic acids, and other phenolic derivatives). Aliphatic groups may be the cause of weak bands at 2922.70 cm^−1^ and 2929.69 cm^−1^ for AgNPs-H_2_O and extract-H_2_O, respectively. Pronounced bands at 1601.42 cm^−1^ and 1608.57 cm^−1^ correspond to C–C bonds in aromatic rings. Other, lower signals, below 1400 cm^−1^, belong to O–H bending, C–C stretching, and C–O stretching. The presence of these bands in the FTIR spectra suggests that in the case of the acetone and water extracts, the chemicals from *A. eupatoria* extracts were involved in the development of a layer that covers the nanoparticles.^47,48^

### 3.3. UV-vis analysis

**Fig. 4:**
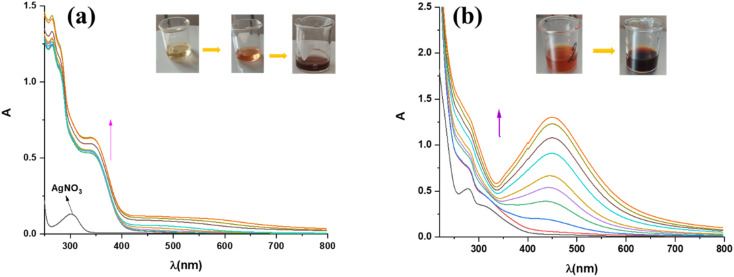
Time-dependent UV-vis absorption spectra (0–3 h) of biosynthesis through the use of (a) the acetone extract of the *Agrimonia eupatoria*, AgNPs-acetone, and (b) the aqueous extract of the *Agrimonia eupatoria*, AgNPs-H_2_O.

The synthesis of silver nanoparticles in the solution, using acetone and aqueous extract of plant *A. eupatoria*, was monitored spectrophotometrically. The hue of the solution changed from light yellow to dark brown (inset in Fig. 4a and b), and the characteristic peak in the UV-vis spectrum is evidence of the formation of AgNPs. This time-dependent process is depicted in (Fig. 4a and b), respectively, for the synthesis of AgNPs-acetone and AgNPs-H_2_O. Indeed, the formation of AgNPs was confirmed by the UV-vis absorption spectra of nanoparticles (300–800 nm), where the most intense peaks were located between 320 nm and 400 nm for AgNPs-acetone and 400 nm and 500 nm for AgNPs-H_2_O. Maximum absorption levels were attained during biosynthesis for three hours, and then the absorption peaks stopped rising, indicating that the process was over. The best conditions for the synthesis of nanoparticles were examined spectrophotometrically. The influence of extract concentration, temperature, pH, and AgNO_3_ concentration on the biogenesis of nanoparticles was monitored. The optimum conditions were found to be by utilizing the acetone extract of the plant after a reaction duration of 3 hours, where the concentration of AgNO_3_ was 5 mM, reaction temperature 25 °C, and pH equals 4.^26^ In the case of aqueous plant extract, the maximum yield of AgNPs was a reaction temperature of 25 °C, pH of 6, reaction duration of 3 h, concentration of 1% extract, and a concentration of 5 mM of AgNO_3_.^27^

### 3.4. Antioxidant activity of extracts and synthesized AgNPs

The antioxidant activity of water and acetone extracts of *A. eupatoria* and synthesized AgNPs was determined by using DPPH and ABTS methods, and the obtained results are presented in Table 1. Comparing the antioxidant activity of aqueous and acetone extracts, as well as their synthesized silver nanoparticles according to the removal of DPPH radical, it can be concluded that AgNPs-acetone exhibited the highest antioxidant capacity with IC_50_ values of 4.6 μg mL^−1^, whereas the IC_50_ of aqueous and acetone extracts is 18.5 and 6.9 μg mL^−1^, respectively. Contrarily, AgNPs-acetone extract showed considerably less antioxidant activity against DPPH radical as compared to the reference antioxidant NDGA (IC_50_ = 0.5 μg mL^−1^). In addition, the antioxidant potential of the examined extracts, as well as their AgNPs, was also checked using the ABTS test and for that purpose as a reference compound Trolox was used (Table 1). The studied extracts showed comparable efficacy against the DPPH and ABTS radicals. Namely, AgNPs-acetone had the greatest antioxidant ability, with IC_50_ values of 5.6 μg mL^−1^, whereas aqueous and acetone extracts had IC_50_ values of 19.9 and 6.5 μg mL^−1^, respectively. The AgNPs-H_2_O was ineffective against DPPH and ABTS radicals.

**Table 1:** The antioxidant activity of the aqueous and acetone extracts of the plant *Agrimonia eupatoria*, and appropriate synthesized AgNPs (AgNPs-acetone and AgNPs-H_2_O)

Investigated samples and standards	IC_50_ values (μg mL^−1^)	
	DPPH˙ scavenging activity	ABTS˙^+^ scavenging activity
Water extract	18.5 ± 0.3	19.9 ± 0.2
Acetone extract	6.9 ± 0.1	6.5 ± 0.1
AgNPs-H_2_O	>100	>100
AgNPs-acetone	4.6 ± 0.1	5.6 ± 0.3
NDGA	0.5 ± 0.1	—
Trolox	—	1.3 ± 0.1

In our study, the examination of the free radical scavenging potency of the extracts and AgNPs toward the DPPH and ABTS radicals revealed that AgNPs-acetone was the best antioxidant, while the AgNPs-H_2_O was ineffective against both radicals. Additionally, very good to moderate activities for investigated extracts was observed towards both radicals. The obtained results indicate that the phenolic and polyphenolic compounds from the extracts, which contribute to the antioxidant potential, are incorporated very efficiently on the surface of the AgNPs-acetone, but not on the surface of the AgNPs-H_2_O. Since, from this investigation, it is evident that both extracts and AgNPs-acetone should be considered very good antioxidants.

### 3.5. Evaluation of DNA damage

Third instar larvae were treated with three different concentrations of *A. eupatoria* water and acetone extracts (0.5, 1, and 2 mg mL^−1^) and DNA damage was measured according to the visual scoring method (Table 2 and Fig. S1†). Based on the results obtained, acetone extract at the tested doses did not significantly increase the frequency of total comet score over the value obtained in the negative control. From Table 2, it is evident that 2 mg mL^−1^ of water extract exposures induced slightly higher level of DNA damage than negative control, although this damage was less than that of the positive control. At the tested concentration of 1 mM, silver nitrate does not result in significant increases in the frequency of total comet score over the value obtained for the negative control (Table 2 group III). Significant increases in total comet scores were observed in the EMS-treated larvae (Table 2 group II). Compared to the values obtained in the positive control, neither of the extract's concentrations tested was able to induce significant increases in total comet score.

**Table 2:** Genotoxic and antigenotoxic effects of different concentrations of the *Agrimonia eupatoria* water and acetone extract^a^

Treatments	Proportion of damaged nuclei (%)					Total comet score^b^	% *R*^c^
	*T*_0_	*T*_1_	*T*_2_	*T*_3_	*T*_4_		
I	68.9 ± 0.51	31.1 ± 0.43	—	—	—	31.1 ± 0.40^e^	—
II	26.1 ± 0.41	36.6 ± 0.25	25.3 ± 0.32	9.9 ± 0.45	2.1 ± 0.51	125.3 ± 0.51^d^^,^^f^	—
III	66.2 ± 0.81	32.4 ± 0.32	1.4 ± 0.60	—	—	35.2 ± 0.25^e^	—
IV	66.5 ± 0.24	33.5 ± 0.47	—	—	—	33.5 ± 0.40^e^	—
V	64.6 ± 0.32	35.4 ± 0.81	—	—	—	35.4 ± 0.80^e^	—
VI	58.9 ± 0.83	39.9 ± 0.54	1.2 ± 0.70	—	—	42.3 ± 0.41^d^^,^^e^^,^^f^	—
VII	67.6 ± 0.23	32.4 ± 0.60	—	—	—	32.4 ± 0.60^e^	—
VIII	66.5 ± 0.80	33.5 ± 0.22	—	—	—	33.5 ± 0.20^e^	—
IX	64.5 ± 0.33	35.5 ± 0.17	—	—	—	35.5 ± 0.17^e^	—
X	45.4 ± 0.72	53.3 ± 0.81	1.24 ± 0.62	—	—	55.8 ± 0.24^d^^,^^e^^,^^f^	73.8
XI	38.9 ± 0.54	59.4 ± 0.61	1.7 ± 0.26	—	—	62.8 ± 0.54^d^^,^^e^^,^^f^	66.3
XII	30.9 ± 0.70	64.8 ± 0.41	3.2 ± 0.52	1.1 ± 0.50	—	74.5 ± 0.32^d^^,^^e^^,^^f^	53.9
XIII	51.5 ± 0.51	46.7 ± 0.41	1.8 ± 0.73	—	—	50.3 ± 0.26^d^^,^^e^^,^^f^	79.6
XIV	45.6 ± 0.25	51.2 ± 0.70	3.2 ± 0.74	—	—	57.6 ± 0.45^d^^,^^e^^,^^f^	71.9
XV	44.7 ± 0.51	51.5 ± 0.71	2.1 ± 0.47	1.7 ± 0.90	—	60.8 ± 0.72^d^^,^^e^^,^^f^	68.5

a I – negative control. II – positive control, ethyl methanesulfonate, EMS, 1 mM; III – silver nitrate, AgNO_3_, 1 mM; IV – water extract of *Agrimonia eupatoria* 0.5 mg mL^−1^; V – water extract of *Agrimonia eupatoria* 1 mg mL^−1^; VI – water extract of *Agrimonia eupatoria* 2 mg mL^−1^; VII – acetone extract of *Agrimonia eupatoria* 0.5 mg mL^−1^; VIII – acetone extract of *Agrimonia eupatoria* 1 mg mL^−1^; IX – acetone extract of *Agrimonia eupatoria* 2 mg mL; X – water extract of *Agrimonia eupatoria* 0.5 mg mL^−1^ + 1 mM EMS; XI – water extract of *Agrimonia eupatoria* 1 mg mL^−1^ + 1 mM EMS; XII – water extract of *Agrimonia eupatoria* 2 mg mL^−1^ + 1 mM EMS; XIII – acetone extract of *Agrimonia eupatoria* 0.5 mg mL^−1^ + 1 mM EMS; XIV – acetone extract of *Agrimonia eupatoria* 1 mg mL^−1^ + 1 mM EMS; XV – acetone extract of *Agrimonia eupatoria* 2 mg mL^−1^ + 1 mM EMS.

b The values are mean ± S.E. from three independent experiments.

c % *R*, percentage of reduction.

d *p* < 0.05 when compared with the negative control group.

e *p* < 0.05 when compared with the positive control group.

f *p* < 0.05 when compared with the silver nitrate group.

The simultaneous treatment was performed to assess the antigenotoxic properties of extracts against EMS-induced DNA damage. The combined treatment with the EMS and 0.5 or 1 mg mL^−1^ water extract revealed the absence of comet classes 3 and 4 and a significant reduction in DNA damage with percentage reductions (% *R*) of 73.8 and 66.3%, respectively, when compared to the EMS-treated larvae (Table 2 groups X and XI *vs.* group II). However, the exposure to EMS and water extract at the highest concentration reduced the EMS-induced DNA damage with a percentage reduction of about 50% (Table 2 group II *vs.* group XII). The combination of EMS and acetone extract (0.5, 1, and 2 mg mL^−1^) significantly decreased EMS-induced DNA damage with percentage reduction levels of 79.6, 71.9, and 68.5% (Table 2 groups XIII, XIV, and XV respectively).

To assess the genotoxicity and antigenotoxicity of biosynthesized AgNPs from the water and acetone extracts of *A. eupatoria*, third instar larvae were treated with three different concentrations, namely 0.5, 1, and 2 mg mL^−1^ for 24 h (Table 3 and Fig. S2†). The exposure to 0.5 mg per mL AgNPs-acetone did not induce statistically significant DNA damage in the third instar larvae (Table 3 group VII). The results indicated that, at the lowest concentration, AgNPs-acetone was not genotoxic. In contrast, 1 and 2 mg per mL AgNPs-acetone and all three tested doses of AgNPs-H_2_O induced a significant increase in the total comet score in relation to the negative control, but this increase was less than the genotoxicity observed in the EMS-treated larvae. The cotreatment with 0.5, 1, and 2 mg mL^−1^ biosynthesized AgNPs-H_2_O plus EMS did not statistically significantly reduce the EMS-induced DNA damage, with values of percentage reduction in DNA damage about 50% (Table 3 groups XI, XI, and XII *vs.* group II). The combination of 0.5, 1, and 2 mg per mL AgNPs-acetone with EMS reduced DNA damage with respect to EMS alone, with percentage reduction levels of 70.3, 64.5, and 61.0 (Table 3 groups XIII, XIV, and XV *vs.* group II respectively).

**Table 3:** Genotoxic and antigenotoxic effects of different concentrations of the silver nanoparticles biosynthesized from water and acetone extracts of *Agrimonia eupatoria* (AgNPs-H_2_O and AgNPs-acetone)^a^

Treatments	Proportion of damaged nuclei (%)					Total comet score^b^	% *R*^c^
	*T*_0_	*T*_1_	*T*_2_	*T*_3_	*T*_4_		
I	68.9 ± 0.51	31.1 ± 0.43	—	—	—	31.1 ± 0.24^e^	—
II	26.1 ± 0.41	36.6 ± 0.25	25.3 ± 0.32	9.9 ± 0.45	2.1 ± 0.92	125.3 ± 0.51^d^^,^^f^	—
III	66.2 ± 0.81	32.4 ± 0.32	1.4 ± 0.60	—	—	35.2 ± 0.25^e^	—
IV	44.4 ± 0.24	40.3 ± 0.21	15.3 ± 0.57	—	—	70.9 ± 0.23^d^^,^^e^^,^^f^	—
V	46.1 ± 0.32	37.5 ± 0.54	16.4 ± 0.31	—	—	70.3 ± 0.34^d^^,^^e^^,^^f^	—
VI	40.7 ± 0.44	44.1 ± 0.91	13.5 ± 0.21	1.7 ± 0.54	—	76.2 ± 0.82^d^^,^^e^^,^^f^	—
VII	69.5 ± 0.20	25.4 ± 0.33	5.1 ± 0.81	—	—	35.6 ± 0.85^e^	—
VIII	64.9 ± 0.41	26.3 ± 0.20	8.8 ± 0.37	—	—	43.9 ± 0.41^d^^,^^e^^,^^f^	—
IX	52.1 ± 0.32	45.1 ± 0.25	2.8 ± 0.50	—	—	50.7 ± 0.20^d^^,^^e^^,^^f^	—
X	33.4 ± 0.60	57.1 ± 0.32	9.5 ± 0.24	—	—	76.1 ± 0.32^d^^,^^e^^,^^f^	52.2
XI	34.6 ± 0.51	51.3 ± 0.53	11.5 ± 0.55	2.6 ± 0.51	—	82.1 ± 0.61^d^^,^^e^^,^^f^	45.9
XII	45.3 ± 0.38	32.1 ± 0.51	13.2 ± 0.34	9.4 ± 0.27	—	86.7 ± 0.82^d^^,^^e^^,^^f^	41
XIII	50 ± 0.82	40.9 ± 0.54	9.1 ± 0.50	—	—	59.1 ± 0.41^d^^,^^e^^,^^f^	70.3
XIV	46.7 ± 0.90	42.1 ± 0.67	11.2 ± 0.94	—	—	64.5 ± 0.32^d^^,^^e^^,^^f^	64.5
XV	40.5 ± 0.39	51.2 ± 0.91	8.3 ± 0.23	—	—	67.8 ± 0.38^d^^,^^e^^,^^f^	61.04

a I – negative control; II – positive control, ethyl methanesulfonate, EMS, 1 mM; III – silver nitrate, AgNO_3_, 1 mM; IV – AgNPs-H_2_O, 0.5 mg mL^−1^; V – AgNPs-H_2_O, 1 mg mL^−1^; VI – AgNPs-H_2_O, 2 mg mL^−1^; VII – AgNPs-acetone, 0.5 mg mL^−1^; VIII – AgNPs-acetone, 1 mg mL^−1^; IX – AgNPs-acetone, 2 mg mL^−1^; X – AgNPs-H_2_O, 0.5 mg mL^−1^ + 1 mM EMS; XI – AgNPs-H_2_O, 1 mg mL^−1^ + 1 mM EMS; XII – AgNPs-H_2_O, 2 mg mL^−1^ + 1 mM EMS; XIII – AgNPs-acetone, 0.5 mg mL^−1^ + 1 mM EMS; XIV – AgNPs-acetone, 1 mg mL^−1^ + 1 mM EMS; XV – AgNPs-acetone, 2 mg mL^−1^ + 1 mM EMS.

b The values are mean ± S.E. from three independent experiments.

c % *R*, percentage of reduction.

d *p* < 0.05 when compared with the negative control group.

e *p* < 0.05 when compared with the positive control group.

f *p* < 0.05 when compared with the silver nitrate group.

Among the different assays used to measure DNA damage and assess the DNA repair of nanomaterials, the comet assay, as one of the most used techniques with many advantages, has already been used to assess the DNA damage caused by a variety nanoparticle through different model systems.^49,50^ The fruit fly *D. melanogaster* is commonly a *in vivo* model organism in studies addressed to determine the potential harmful effects of nanomaterials, including AgNPs.^32,51–53^ In the current study, the genotoxicity and antigenotoxicity of different concentrations of *A. eupatoria* water and acetone extracts and biosynthesized AgNPs from extracts were evaluated in the anterior midguts of third instar larvae of *D. melanogaster* by alkaline comet assay. The observed activities were compared with those obtained by silver nitrate, as an agent acting *via* the release of silver ions. Only a few *in vivo* studies have been reported using the comet assay and/or *D. melanogaster* as model organism to determine the genotoxicity of commercial AgNPs or photosynthesized AgNPs.^54–56^ Previously, the genotoxic effects of commercial AgNPs and silver nitrate in *D. melanogaster* hemocytes have been determined by comet assay.^32^ In *D. melanogaster* wings imaginal disks AgNPs were also induced significant levels of genotoxic damage as determined by the wing-spot assay.^51^ In our results it was noticed that AgNPs-acetone induced a milder genotoxic potential compared to AgNPs-H_2_O in the tested concentration range. To the best of our knowledge, *A. eupatoria* extracts or the AgNPs synthesized from *A. eupatoria* extracts have not been tested using the comet assay or *D. melanogaster* as an *in vivo* model organism.

### 3.6. Hemolytic activity

The effects of synthesized nanoparticles, corresponding plant extracts and AgNO_3_ in different concentrations on the release of hemoglobin from treated erythrocytes were spectrophotometry monitored at 540 nm followed by one-hour incubation at 37 °C. The obtained results are shown in Table 4. In the present study, the negative control (PBS only) was used as a reference value for non-induction of hemolysis (0%), while the positive control (with 1% SDS) was used as a reference value for fully induced hemolysis (100%). Based on literature data, the hemolytic activity of a drug that causes 2% or less hemolysis is considered nonhemolytic, above 5% hemolysis, the hemolytic activity is considered significant, while from 2 to 5% hemolysis, the hemolytic activity of the drug is considered slightly.^57^ The results shown in Table 4 indicate that none of the tested concentrations of H_2_O extract of *A. eupatoria* exhibited any hemolytic activity. The acetone extract showed slight hemolytic activity on rat erythrocytes in a dose-dependent manner from a dose of 30 μg mL^−1^ up to 90 μg mL^−1^, and significant activity for the two highest applied doses (130 and 150 μg mL^−1^). Lower doses (1 and 10 μg mL^−1^) of acetone extract of *A. eupatoria* didn't affect the erythrocytes membrane. Lower doses of AgNO_3_ also didn't affect hemoglobin release, up to the dose of 90 μg mL^−1^, which caused slight hemolysis, while the two highest applied doses displayed significant hemolytic activity. Regarding silver nanoparticles, newly synthesized AgNPs-H_2_O and AgNPs-acetone exhibited significant hemolytic activity in a dose-dependent manner from the concentration of 30 μg mL^−1^ up to the highest applied concentration. Concentrations of 1 and 10 μg mL^−1^ of both examined nanoparticles do not influence the erythrocytes membrane or induce slight hemolysis (10 μg per mL AgNPs-H_2_O).

**Table 4:** Hemolytic activity of AgNPs (AgNPs-H_2_O and AgNPs-acetone), *Agrimonia eupatoria* water and acetone extracts, and AgNO_3_^a^

Concentrations (μg mL^−1^)	% hemolysis				
	AgNPs-H_2_O	AgNPs-acetone	H_2_O extract	Acetone extract	AgNO_3_
150	89.35 ± 0.07	85.31 ± 0.13	<negative control	11.50 ± 0.78	36.25 ± 1.41
120	88.11 ± 0.53	73.97 ± 0.68	<negative control	11.03 ± 0.78	20.28 ± 1.21
90	84.61 ± 0.94	60.92 ± 1.01	<negative control	4.89 ± 0.40	3.96 ± 1.75
60	69.11 ± 1.68	42.66 ± 1.21	<negative control	4.74 ± 0.78	1.51 ± 1.01
30	26.11 ± 0.81	20.75 ± 1.61	<negative control	4.66 ± 0.13	<negative control
10	2.91 ± 0.20	<negative control	<negative control	0.46 ± 1.61	<negative control
1	<negative control	<negative control	<negative control	<negative control	<negative control

a Experiment were performed in triplicate. Data are presented as the mean ± S.E.

Since systemic circulation is the primary route of distribution of xenobiotics to target tissues, erythrocytes are the first cells that can be affected by the potential negative effects of the applied substances. Due to the lack of organelles and nuclei, the recovery of erythrocytes from injury is limited.^58^ For further investigations, it is extremely important to examine the hemolytic activity of newly synthesized substances. Because of the ease and speed of performing the hemolytic test, it is widely used, and its results provide a good estimate of the toxicity of the tested treatments. For possible future medical use, it is of great importance that nanoparticles do not have or have minimal hemolytic activity.^59^ A potential decrease in the number of erythrocytes due to slight hemolysis, for healthy mammalian species, will be resolved by *de novo* synthesis and release of circulating reticulocytes.^60^ Based on the results obtained in this study, both used extracts and AgNO_3_ in almost all tested concentrations, except the two highest (120 μg mL^−1^ and 150 μg mL^−1^), which didn't significantly affect the erythrocytes membrane. On the other hand, treatment with different concentrations of AgNPs synthesized from H_2_O and acetone extract of *A. eupatoria* mainly induced a significant percentage of hemolysis, which correspondingly increased with the increase of concentration of the tested nanoparticles. The lowest concentration of both AgNPs which induced over 20% of hemolysis was 30 μg mL^−1^, so it could be concluded that all the higher tested concentrations, including the mentioned one, exhibited toxic effects on the erythrocytes. Only two concentrations, where the hemolytic activity of tested AgNPs was not registered, or it was slightly, were the concentrations of 1 μg mL^−1^ and 10 μg mL^−1^. These concentrations of examined AgNPs do not affect the erythrocyte's fragility and could be considered safe for use in further analysis. Our findings are in correspondence with the other studies where it was demonstrated that some of the green-synthetized silver nanoparticles could induce hemolysis of erythrocytes in a dose-dependent manner.^61,62^ Furthermore, it was demonstrated that lower doses (up to 5 μg mL^−1^) of AgNPs synthesized by using *Catharanthus roseus* didn't affect the stability of erythrocytes membrane, while the higher doses (10 μg mL^−1^, 25 μg mL^−1^, and 50 μg mL^−1^) exhibited a toxic effect on this cells^61,63^ concluded that hemolysis of erythrocytes induced by AgNPs could be due to the direct Ag^+^ and membrane interaction, thus forming reactive species and oxidative stress. Moreover, the hemolytic activity of the compounds could be variable and dependent on such factors as temperature, incubation period as well the composition of the plant extract.^62^

### 3.7. Anticancer activity of AgNPs

#### 3.7.1 Cytotoxic activity

**Fig. 5:**
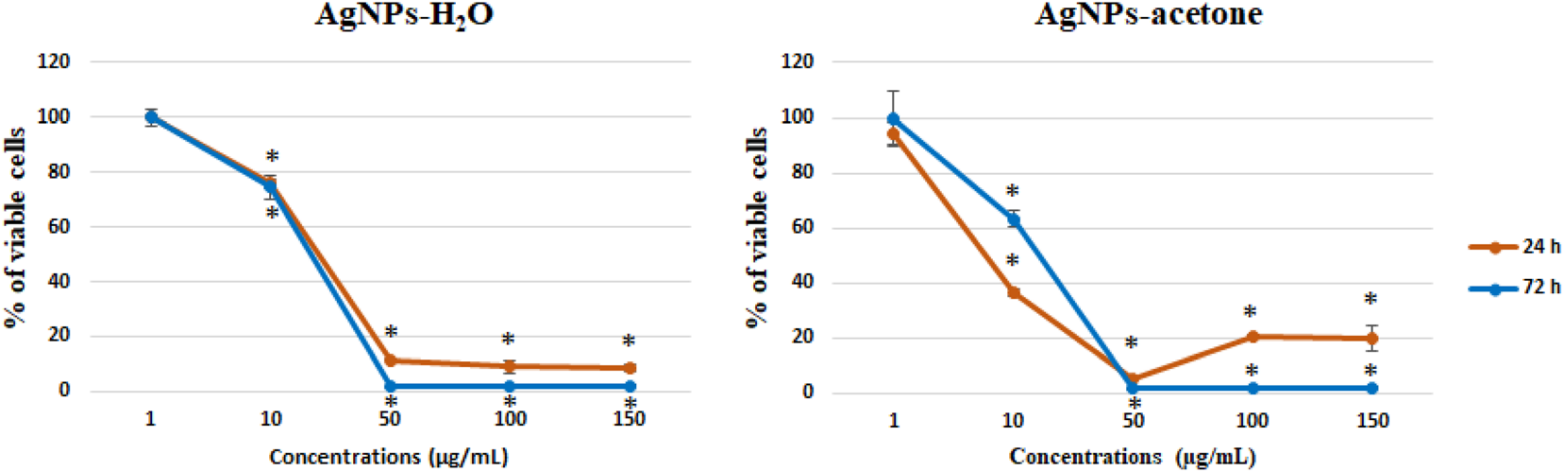
Effect of AgNPs-H_2_O and AgNPs-acetone on SW-480 cells viability, 24 and 72 h after treatment.

Cytotoxic activity of silver nanoparticles water (AgNPs-H_2_O) and acetone extract (AgNPs-acetone) on SW-480 cells viability was presented in Fig. 5. The viability of SW-480 colorectal cancer cells was significantly decreased under the influence of treatments after 24 h and 72 h, about control. Based on the cell viability curve, IC_50_ values, as a parameter of cytotoxicity, were calculated. Observed values indicate high and time-dependent cytotoxicity of these treatments on SW-480 cancer cells (Table 5). Better cytotoxicity, with lower IC_50_ values, was observed in treatment by AgNPs-acetone extract.

**Table 5:** The cytotoxic effect of AgNPs-H_2_O and AgNPs-acetone expressed as IC_50_ value (μg mL^−1^) on SW-480 cells^a^

Cell line	AgNPs-H_2_O (μg mL^−1^)		AgNPs-acetone (μg mL^−1^)	
	24 h	72 h	24 h	72 h
SW-480	32.35 ± 1.25	17.34 ± 0.58	18.36 ± 0.17	15.84 ± 0.18

a The results are presented as the mean ± standard error of three independent experiments.

Nanotechnology also plays a very important role in antitumor therapy and the establishment of targeted therapies. Frequent problems related to the limitations of commercial cytostatic indicate the need to develop more natural drugs that will not have a wide range of side effects, where the research of nanocomposites is of great importance. Specifically, silver nanoparticles have already been described in the literature as an antitumor agent with cytotoxic and proapoptotic effect.^64–66^ In this paper, the anticancer activity of AgNPs-H_2_O and AgNPs-acetone obtained by reduction of silver nitrate with reduction extracts of the plant *A. eupatoria* was demonstrated for the first time. Our results show a significant decrease in SW-480 cell viability by the silver nanoparticles AgNPs-H_2_O and AgNPs-acetone extracts synthetized using plant species *A. eupatoria*. The low IC_50_ values obtained indicate strong cytotoxicity and potential for future detailed analysis. Besides the silver nanoparticles, some authors have reported that even the extracts of *A. eupatoria* induce noticeable cytotoxicity on cancer cell lines after various incubation periods.^67^ On the other hand, in recent years there is the rapid synthesis of nanoparticles using biological (plants, fungi, algae, and bacteria) techniques, where many possess significant antitumor potential, like carboxymethyl cellulose and silver nanoparticles (CMC-AgNPs) on liver (HepG2) cancer cell line,^68^ AgNPs on cervical cancer cells,^69^ and exhibited cytotoxicity towards different cancer cell lines (U-87, MCF-7, HeLa, PANC-1 and B16F10).^70^

#### 3.7.2 Proapoptotic activity

**Fig. 6:**
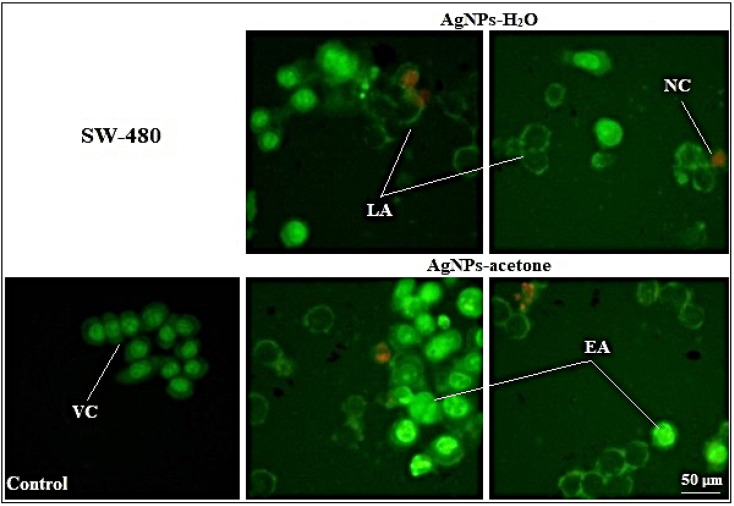
Micrographs from the fluorescent microscope (400× magnification). Morphology of SW-480 control cells and cells treated with AgNPs-H_2_O and AgNPs-acetone in IC_50_ concentration (μg mL^−1^). Viable cells-VC; early apoptosis-EA; late apoptosis-LA and necrotic cells-NC.

The determination of the type of induced cell death by AgNPs shows the significant occurrence of apoptosis under the influence of both silver nanoparticle extracts, in comparison with untreated cells. In the treatment of AgNPs-H_2_O, all detected apoptotic cells were in the late stage of apoptosis, while in the treatment of AgNPs-acetone, the early stage of apoptosis was also detected (20.01 ± 1.11%). Cells in necrosis were also detected but in a smaller percentage (Table 6), where the AgNPs-H_2_O induced higher percentage of necrosis compared to AgNPs-acetone. The typical morphological changes characteristic for early and late stages of apoptosis, and necrosis in AgNPs treated cells stained by AO/EB were detectable on micrographs (Fig. 6).

**Table 6:** The percentages of viable, early apoptotic, late apoptotic, and necrotic cells in the total number of controls SW480 cells and cells treated by AgNPs-H_2_O and AgNPs-acetone extract on SW-480 cells, 24 h after applied treatment^a^

	μM	Viable cells	Early apoptosis	Late apoptosis	Necrosis
Control	0	95.41 ± 3.41	3.9 ± 0.09	0.69 ± 0.01	—
AgNPs-H_2_O	IC_50_	15.46 ± 0.52*	0.52 ± 0.02*	69.38 ± 1.14*	14.64 ± 1.13*
AgNPs-acetone	IC_50_	16.17 ± 0.91*	20.01 ± 1.11*	53.93 ± 1.41*	9.89 ± 0.21*

a The results are presented as the mean ± standard error of three independent experiments. *Statistically significant difference (*p* < 0.05) compared to control values.

Obtained results indicate that the cytotoxic activity of AgNPs-H_2_O and AgNPs-acetone was through induction of apoptosis, which was confirmed by AO/EB cell staining. Some authors also prove the pro-apoptotic activity of silver nanoparticles by studying their mechanism, which indicates an increase in the expression of initiator caspases 8 and 9 in cancer cell lines.^59,60^ Also, silver nanoparticles synthesized using *Acorus calamus* rhizome show excellent antitumor activity on Hep2, COLO 205 and SH-SY5Y, while Hep2 cells were the most sensitive and proapoptotic activity was also proven on them supposed through an increase of ROS.^71^ Other authors have reported combination and synergistic interactions of AgNPs and camptothecin, where they cause cell death by inducing the mitochondrial membrane permeability change and activation of caspase 9, 6, and 3. The proapoptotic effect seems to also be associated with increased ROS formation.^69^

The biological activity may depend on particle size. The result of antitumor activity shows a better activity of AgNPs-acetone (35 nm) than AgNPs-H_2_O (40 nm). Also, the higher percentage of induced necrosis with AgNPs-H_2_O may be the result of the inability of the particles to enter the cells through cell membrane, or mechanically induced necrosis. Considering our results and a review of previous literature data of other authors, silver nanoparticles show good potential for further detailed investigation to use them in anticancer therapy.

### 3.8. Antimicrobial activity

The results of *in vitro* antibacterial and antifungal activities of water and acetone nanoparticles, determined by MICs and MMCs, are shown in Tables 7 and 8. A total of 23 species of microorganisms were tested and the results were compared with the influence of ampicillin and tetracycline for bacteria, and with itraconazole and amphotericin B for fungi. Tetracycline is a broad-spectrum antibiotic effective against aerobic and anaerobic G^−^ and G^+^ bacteria. Ampicillin is a beta-lactam antibiotic that inhibits the growth of G^+^ and some G^−^ bacteria. Amphotericin B is an antifungal drug often used for serious systemic fungal infections and is one of the most effective treatments for fungal infections caused by *Aspergillus* sp. Itraconazole prevents the growth of *Candida* sp.

**Table 7:** Antibacterial activity of AgNPs-H_2_O and AgNPs-acetone^a^

Bacteria	AgNPs-H_2_O		AgNPs-acetone		Tetracycline		Ampicillin	
	MIC^b^	MBC^b^	MIC^b^	MBC^b^	MIC^c^	MBC^c^	MIC^c^	MBC^c^
*Escherichia coli* ATCC 25922	0.156	0.156	0.625	0.625	4	6	0.37	0.5
*Pseudomonas aeruginosa* ATCC 27853	0.156	0.625	0.3125	0.625	4	32	>128	>128
*E. coli*	0.3125	0.625	1.25	2.5	2	6	2.1	1.2
*Salmonella enterica*	0.3125	0.625	1.25	2.5	2	2	1	1
*S. typhimurium*	0.3125	0.625	0.625	1.25	2	2	2	2
*P. aeruginosa*	0.3125	0.625	0.3125	0.625	>128	>128	>128	>128
*Proteus mirabilis*	0.156	0.625	0.625	1.25	>128	>128	>128	>128
*K. pneumoniae*	0.625	2.5	0.625	1.25	4	32	>128	>128
*Staphylococcus aureus* ATCC 25923	0.078	0.156	0.156	0.156	1.5	3	0.25	0.75
*Bacillus subtilis* ATCC 6633	0.625	1.25	0.156	0.625	0.25	0.37	3	4
*Enterococcus faecalis* ATCC 29212	0.3125	1.25	0.3125	0.625	8	12	1	2
*S. aureus*	0.625	1.25	0.3125	0.625	<0.06	<0.06	<0.06	<0.06
*B. subtilis*	1.25	>5	0.625	1.25	<0.06	0.25	16	128
*B. cereus*	0.625	2.5	0.3125	0.625	0.25	0.5	4	6
*E. faecalis*	0.039	0.078	0.156	0.155	1	6	4	6
*Lactobacillus rhamnosus*	0.625	0.625	0.625	1.25	0.16	1	64	13
*Bifidobacterium animalis* subsp. *lactis*	0.078	0.078	0.078	0.078	4	8	<0.06	12

a MIC – minimum inhibitory concentration; MBC – minimum bactericidal concentration.

b MIC and MBC values are given as mg mL^−1^.

c MIC and MBC values are given as μg mL^−1^.

**Table 8:** Antifungal activity of AgNPs-H_2_O and AgNPs-acetone^a^

Fungi	AgNPs-H_2_O		AgNPs-acetone		Amphotericin B		Itraconazole	
	MIC^b^	MFC^b^	MIC^b^	MFC^b^	MIC^c^	MFC^c^	MIC^c^	MFC^c^
*Candida albicans* ATCC 10231	0.625	5	0.625	5	0.49	1.95	1.95	1.95
*C. albicans*	0.625	5	0.625	5	0.98	1.95	1.95	1.95
*Penicillium italicum*	0.078	0.078	0.078	0.078	7.81	7.81	0.98	0.98
*P. chrysogenum*	0.078	0.078	0.078	0.078	7.81	7.81	0.98	0.98
*Aspergillus flavus*	0.625	1.25	1.25	2.5	0.98	15.62	0.98	0.98
*A. niger*	1.25	2.5	1.25	2.5	0.98	0.98	15.62	15.62

a MIC – minimum inhibitory concentration; MFC – minimum fungicidal concentration.

b MIC and MFC values are given as mg mL^−1^.

c MIC and MFC values are given as μg mL^−1^.

In this research paper, the values of the MIC and MMC were in the range of 0.078 mg mL^−1^ to 5 mg mL^−1^. The intensity of antibacterial activity was found to be dependent on the type of nanoparticles and the species of bacteria. The antibacterial activity of silver nanoparticles was tested on 8 Gram-negative (Fig. S3 and S4†) and 9 Gram-positive (Fig. S5–S7†) bacteria, and the activity was equally good on G^+^ and G^−^ bacteria. AgNPs-H_2_O showed activity at a lower concentration than AgNPs-acetone, as seen in the MIC values against *E. coli* ATCC 25922, *P. aeruginosa* ATCC 27853, *E. coli*, *S. enterica*, *S. typhimurium*, *P. mirabilis*, *S. aureus* ATCC 25923, and *E. faecalis*. The MBC values of AgNPs-H_2_O for the mentioned species were less than or the same as the MBC of AgNPs-acetone. MIC and MBC values of AgNPs-H_2_O and AgNPs-acetone were the same for *P. aeruginosa*, while the MIC value of AgNPs-H_2_O and AgNPs-acetone was the same for *K. pneumoniae* and *E. faecalis* ATCC 29212, but MBC values were different for that species. The MIC value of AgNPs-H_2_O was lower than the MIC of AgNPs-acetone against *P. mirabilis*. The MIC and MBC values of AgNPs-acetone were at lower concentrations than those of AgNPs-H_2_O against the species *B. subtilis* ATCC 6633, *S. aureus*, *B. subtilis*, and *B. cereus*. MBC value of AgNPs-H_2_O was >5 against *B. subtilis*. The MIC and MBC values of AgNPs-H_2_O were lower against *E. faecalis* than MIC and MBC values of AgNPs-acetone. MIC values of AgNPs-H_2_O and AgNPs-acetone were the same for *L. rhamnosus* and *B. animalis* subsp. *lactis*, while the MBC value differed only against *L. rhamnosus*.

Antifungal activity of AgNPs-H_2_O and AgNPs-acetone was tested on yeast and filamentous fungi. MIC values of AgNPs-H_2_O and AgNPs-acetone were the same for all fungi except for species *A. flavus*, where the MIC of AgNPs-was lower (Fig. S8†). The MFC values were also the same, except for the species *A. flavus*, where the MFC value of AgNPs-H_2_O was lower.

In this paper, for the first time, the antimicrobial activity of AgNPs-H_2_O and AgNPs-acetone obtained by reduction of silver nitrate with extracts of the plant *A. eupatoria* was demonstrated. Based on the results, it can be concluded that the antimicrobial activity of the obtained nanoparticles is at low concentrations and that they act on G^−^ and G^+^ bacteria. In comparison with the antimicrobial activity of *A. eupatoria* extracts, the obtained nanoparticles of aqueous and acetone extracts show an equally good antimicrobial effect both on G^−^ and on G^+^ bacteria, which is not the case with the examined extracts. No bacterial strain showed resistance to the tested nanoparticles. Based on the results, it is concluded that the use of *A. eupatoria* extract for the purpose of obtaining nanoparticles is justified considering that the nanoparticles show good antibacterial activity without the appearance of resistance. This is especially important because the resistance of the G^−^ bacteria could be attributed to their cell wall structure. G^−^ bacteria have an effective permeability barrier composed of a thin lipopolysaccharide exterior membrane, which could restrict the penetration of the active compounds from plant extracts.^72^ The MIC values of AgNPs-H_2_O and AgNPs-acetone were the same for all fungi except for the species *A. flavus* where the MIC was lower with AgNPs-H_2_O. The MMC values were also the same except for the species *A. flavus* where the MMC value was lower with AgNPs-H_2_O. Regarding the results given by Muruzović *et al.*^17^ and regarding the effect of the extract on fungi, it is concluded that nanoparticles are effective both on yeasts and on filamentous fungi. The examined fungi showed low sensitivity to the tested extracts.

The antibacterial activity of silver has been well known since ancient times^73–79^ demonstrating that, in low concentrations, silver is nontoxic to human cells. With the increase of microbial resistance to multiple antibiotics many researchers have tried to develop new, effective antimicrobial agents free of resistance and cost. Silver in the form of nanoparticles is a promising candidate for the development of future antibacterial therapies because of its wide spectrum of activity. Xia *et al.*^77^ have demonstrated that AgNPs destroy the cell wall, infiltrate within the cells, damage organelles, and induce chromatin condensation and margination, a sign of apoptotic cell death. Also, it has been confirmed that the size of nanoparticles influences their antimicrobial activity, smaller-sized nanoparticles pass unhindered through the cell membrane, expressing their activity within the cell. According to reported studies, the antimicrobial activity of AgNPs is also attributed to the electrostatic interaction between positively charged silver ions and negatively charged cell walls.^79^ Due to a slightly different cell membrane of the fungi composed mostly of fibrous β-1,3-glucan and mannoproteins, interaction with AgNPs is quite different.^79^ This fact may be one of the causes for the slightly lower antifungal activity of obtained AgNPs compared with their antibacterial activity in some cases. The research conducted by Bhagat *et al.* (2018)^18^ indicated that silver nanoparticles synthesized by chemical reaction using an aqueous extract of *R. brunonii* as a reducing agent showed a concentration-dependent antibacterial activity against the *E. coli*, *K. pneumoniae*, and *B. cereus*. According to Martinez-Castanon *et al.*^80^ MIC values were obtained for the synthesized nanoparticles tested against *E. coli* ATCC 25922 and *S. aureus* ATCC 25923. In this research, the cellular wall content plays an important role in these results. The authors noticed that for *E. coli*, there is no significant difference between the MIC of 29 nm and 89 nm silver nanoparticles. MIC values were lower for G^−^ bacteria than G^+^, suggesting that cell wall thickness was of key importance. They also conclude that with 10 nm silver nanoparticles, there is no significant difference in the MIC against each bacterium. These results are consistent with the results of our research because AgNPs-H_2_O and AgNPs-acetone also showed a better antibacterial effect on some G^−^ strains than on G^+^. The intensity of antibacterial activity was found to be dependent on the type of nanoparticles and the species of bacterial.^8,81^ The average size of the obtained nanoparticles is ∼35 nm for AgNPs-H_2_O and 20–50 nm for AgNPs-acetone.

## 4. Conclusion

Considering the higher potential of using nanoparticles, in this research, we tried to show their potential application through different types of testing. Numerous studies have demonstrated that *Agrimonia eupatoria* is a crucial source of various biologically active compounds, with some of its extracts exhibiting significant antioxidant potential.^25,82–86^ In addition, many studies have reported that the nanoparticles obtained from different plant extracts have a higher antioxidant potential than the extract used for their synthesis, but there is also some research with opposite results.^21,87,88^

This comprehensive study investigated the synthesis and properties of silver nanoparticles (AgNPs) utilizing *A. eupatoria* extracts, specifically acetone and aqueous extracts. Advanced characterization techniques, including TEM, STEM-HAADF imaging, and FTIR analysis, provided detailed insights into the morphology, crystalline structure, and chemical composition of the nanoparticles. The research elucidated optimal synthesis conditions and kinetics, shedding light on the formation process. Evaluation of antioxidant potential revealed that AgNPs-acetone exhibited superior radical scavenging compared to the corresponding plant extract, suggesting promising applications in antioxidant therapies. It was observed that AgNPs-acetone induced a milder genotoxic potential compared to AgNPs-H_2_O in the tested concentration range, while co-treatment with AgNPs-acetone and ethyl methane sulfonate showed a significant reduction of DNA damage caused by this mutagen with a percentage reduction of 70.3%. Concentration-dependent safety considerations were highlighted in hemolytic potential evaluations for both extracts and AgNPs. AgNPs-H_2_O also exhibited more intense hemolytic activity compared to AgNPs-acetones. The lowest concentrations (1 μg mL^−1^ and 10 μg mL^−1^) of both examined (used) nanoparticles had no hemolytic effect on erythrocytes and therefore can be marked as non-toxic and suitable for future research and potential medical application. The multifaceted activities of AgNPs, including cytotoxic, proapoptotic, antibacterial, and antifungal effects, underscored their potential in cancer therapy and antimicrobial interventions. Briefly, AgNPs-acetones and AgNPs-H_2_O induce significant cytotoxic and proapoptotic activity on tumor cells, where AgNPs-acetones show better cytotoxicity, as well as a lower percentage of induced necrosis compared to AgNPs-H_2_O. Regarding the antimicrobial effect of the tested nanoparticles, good antimicrobial activity was observed against G^−^ and G^+^ bacteria at low concentrations, which is very important due to the problem of the emergence of resistance to antibiotics, especially G^−^ bacteria.

This research provides a comprehensive understanding of AgNPs, laying the groundwork for diverse applications. Future investigations should focus on elucidating variations in antioxidant activity between AgNPs-acetone and AgNPs-H_2_O, exploring nanoparticle size and concentration effects on genotoxicity, and understanding the protective mechanisms of *A. eupatoria* extracts. Further research into synergistic interactions between nanoparticles and extracts, the concentration-dependent impact on hemolysis, and the molecular basis of cytotoxicity on tumor cells is recommended. Additionally, exploring antimicrobial properties at lower concentrations, where no hemolytic activity was observed, could reveal insights into their safe and effective use. A systematic exploration of these aspects will refine the applications and safety considerations of *A. eupatoria*-synthesized AgNPs.

## Author contributions

Katarina Marković: investigation, validation, Ana Kesić: supervision, validation, writing – review & editing, Mirjana Novaković: investigation, methodology, validation, Mirjana Grujović: investigation, methodology, Dušica Simijonović: investigation, validation, Edina H. Avdović, investigation, methodology, Sanja Matić: investigation, validation, Milica Paunović: investigation, methodology, Milena Milutinović: investigation, validation, Danijela Nikodijević: investigation, methodology, Olgica Stefanović; investigation, validation and Zoran Marković: data curation, investigation, methodology.

## Conflicts of interest

There are no conflicts to declare.

## Supplementary Material

RA-014-D3RA07819A-s001

## References

[cit1] Iravani S. (2011). Green Chem..

[cit2] Meyers M. A., Mishra A., Benson D. J. (2006). Prog. Mater. Sci..

[cit3] Mahajan P., Verma S., Padha B., Ahmed A., Arya S. (2023). J. Alloys Compd..

[cit4] Dubey A., Singh A., Sharma A., Sundramoorthy A. K., Mahadeva R., Gupta V., Dixit S., Arya S. (2023). Appl. Phys..

[cit5] Sharma A., Arya S., Singh B., Prerna A. T., Singh S., Sharma R. (2020). Integr. Ferroelectr..

[cit6] Rana S. B., Singh R. P. P., Arya S. (2017). J. Mater. Sci.: Mater. Electron..

[cit7] Thakkar K. N., Mhatre S. S., Parikh R. Y. (2010). Nanomed. Nanotechnol. Biol. Med..

[cit8] Gahlawat G., Choudhury A. R. (2019). RSC Adv..

[cit9] Soni M., Mehta P., Soni A., Goswami G. K. (2018). Journal of Biotechnology and Biochemistry.

[cit10] Pal G. , Rai P. and Pandey A., Green Synthesis, Characterization and Applications of Nanoparticles, Elsevier, 2019, pp. 1–26

[cit11] Roychoudhury A. (2020). Indian J. Pharm. Biol. Res..

[cit12] Bahrulolum H., Nooraei S., Javanshir N., Tarrahimofrad H., Mirbagheri V. S., Easton A. J., Ahmadian G. (2021). J. Nanobiotechnol..

[cit13] Zeng W., Tang X., Wu T., Han B., Wu L. (2023). Anal. Chim. Acta.

[cit14] Wu L., Yan H., Li H., Xu X., Zhu L., Chen X., Wang J. (2019). Food Anal. Methods.

[cit15] Wang P., Wu L., Lu Z., Li Q., Yin W., Ding F., Han H. (2017). Anal. Chem..

[cit16] Yin W., Wu L., Ding F., Li Q., Wang P., Li J., Han H. (2018). Sens. Actuators, B.

[cit17] Muruzović M. Ž., Mladenović K. G., Stefanović O. D., Vasić S. M., Čomić L. R. (2023). J. Food Drug Anal..

[cit18] Bhagat M., Anand R., Datt R., Gupta V., Arya S. (2019). J. Inorg. Organomet. Polym. Mater..

[cit19] Oliver S., Wagh H., Liang Y., Yang Sh., Boyer C. (2018). J. Mater. Chem. B.

[cit20] Yadav A., Kon K., Kratosova G., Duran N., Ingle A. P., Rai M. (2015). Biotechnol. Lett..

[cit21] Sondi I., Salopek-Sondi B. (2004). J. Colloid Interface Sci..

[cit22] Srećković N. Z., Nedić Z. P., Liberti D., Monti D. M., Mihailović N. R., Katanić Stanković J. S., Dimitrijević S., Mihailović V. P. (2021). RSC Adv..

[cit23] Srećković N. Z., Nedić Z. P., Monti D. M., D'Elia L., Dimitrijević S. B., Mihailović N. R., Katanić Stanković J. S., Mihailović V. B. (2023). Molecules.

[cit24] Sharma V. K., Yngard R. A., Lin Y. (2009). Adv. Colloid Interface Sci..

[cit25] Srikar S. K., Giri D. D., Pal D. B., Mishra P. K., Upadhyay S. N. (2016). Green Sustainable Chem..

[cit26] Marković G. K. , Grujović Ž. M., Kesić A. S. and Markovic Z. S., 8th Edition of Our Series of International Electronic Conferences on Medicinal Chemistry, (ECMC 2022), MDPI, 2022

[cit27] Kesić A. S., Marković K. G., Grujović M. Ž., Markovic Z. S. (2023). Materials Proceedings.

[cit28] Rasband W. S. , ImageJ, U. S. National Institutes of Health, Bethesda, Maryland USA, https://imagej.nih.gov/ij/

[cit29] Antonijević N. R., Simijonović D. M., Avdović E. H., Ćirić A., Petrović Z. D., Dimitrić Marković J., Stepanić V., Marković Z. S. (2021). Antioxidants.

[cit30] Pontiki E., Hadjipavlou-Litina D., Litinas K., Geromichalos G. (2014). Molecules.

[cit31] Carmona E. R., Guecheva T. N., Creus A., Marcos R. (2011). Environ. Mol. Mutagen..

[cit32] Alaraby M., Romero S., Hernández A., Marcos R. (2019). Environ. Mol. Mutagen..

[cit33] Howell S. l., Taylor K. W. (1968). Biochem. J..

[cit34] Mukhopadhyay I., Chowdhuri D. K., Bajpayee M., Dhawan A. (2004). Mutagenesis.

[cit35] Siddique H. R., Chowdhuri D. K., Saxena D. K., Dhawan A. (2005). Mutagenesis.

[cit36] Singh N. P., McCoy M. T., Tice R. R., Schneider E. L. (1988). Exp. Cell Res..

[cit37] Collins A. R. (2004). Mol. Biotechnol..

[cit38] Manoharan K., Banerjee M. R. (1985). Cell Biol. Int. Rep..

[cit39] Waters N. D., Brady A. L., Stack H. F., Brockman H. E. (1990). Mutat. Res..

[cit40] Mosmann T. (1983). J. Immunol. Methods.

[cit41] Nikodijević D., Jovankić J., Cvetković D., Anđelković M., Nikezić A., Milutinović M. (2021). Eur. J. Pharmacol..

[cit42] Baskić D., Popović S., Ristić P., Arsenijević N. N. (2006). Cell Biol. Int..

[cit43] Andrews J. M. (2005). J. Antimicrob. Chemother..

[cit44] Sarker S. D., Nahar L., Kumarasamy Y. (2007). Methods.

[cit45] Balážová L., Wolaschka T., Rohaľová S., Daneu N., Stahorský M., Salayová A., Tkáčiková L., Eftimová J. (2023). Life.

[cit46] Salayová A., Bedlovičová Z., Daneu N., Baláž M., Lukáčová Bujňáková Z., Balážová L., Tkáčiková L. (2021). Nanomaterials.

[cit47] Konai N., Raidandi D., Pizzi A., Meva'a M. (2017). Eur. J. Wood Wood Prod..

[cit48] Gecer E. N. (2021). J. Inorg. Organomet. Polym. Mater..

[cit49] Kaygisiz S. Y., Ciğerci I. H. (2017). Toxicol. Ind. Health.

[cit50] Liman R., Ali M. M., Istifi E. S., Ciğerci I. H., Bonciu E. (2022). Environ. Sci. Pollut. Res..

[cit51] Demir E., Vales G., Kaya B., Creus A., Marcos R. (2011). Nanotoxicology.

[cit52] Panacek A., Prucek R., Safarova D., Dittrich M., Richtrova J., Benickova K., Zboril R., Kvitek L. (2011). Environ. Sci. Technol..

[cit53] Alaraby M., Annangi B., Marcos R., Hernández D. (2016). J. Toxicol. Environ. Health, Part B.

[cit54] Heikal Y. M., Şuţan N. A., Rizwan M., Elsayed A. (2020). Chemosphere.

[cit55] Moteriya P., Chanda S. (2020). J. Inorg. Organomet. Polym. Mater..

[cit56] Alkan H., Ciğerci I. H., Ali N. M., Hazman O., Liman R., Colă F., Bonciu E. (2022). Plants.

[cit57] Ain Q. U., Munir H., Jelani F., Anjum F., Bilal M. (2020). Mater. Res. Express.

[cit58] Liao C., Li Y., Tjong S. (2019). Int. J. Mol. Sci..

[cit59] Greco L., Molchanova N., Holmedal E., Jenssen H., Hummel B. D., Watts J. L., Håkansson J., Hansen P. R., Svenson J. (2020). Sci. Rep..

[cit60] Behling-Kelly E. (2022). Toxicol. Pathol..

[cit61] Raja A., Salique S. M., Gajalakshmi P., James A. (2016). Int. J. Pharm. Sci. Nanotechnol..

[cit62] Andleeb S., Tariq F., Muneer A., Nazir T., Shahid B., Latif Z., Abbasi G. A., Haq I. U., Majeed Z., Khan S. U., Khan T. M., Al Farraj D. A. (2020). Green Process. Synth..

[cit63] Huang H., Lai W., Cui M., Liang L., Lin Y., Fang Q., Liu Y., Xie L. (2016). Sci. Rep..

[cit64] Kajani A. A., Bordbar A., Esfahani H. Z., Khosropour A. R., Razmjoua A. (2014). RSC Adv..

[cit65] Anandan M., Poorani G., Boomi P., Varunkumar K., Anand K., Chuturgoon A. A., Saravanan M., Gurumallesh N. P. (2019). Process Biochem..

[cit66] Salem S. S., Hashem A. H., Sallam A. M., Doghish A. S., Al-Askar A. A., Arishi A. A., Shehabeldine A. M. (2022). Polymers.

[cit67] Ad’hiah A. H., Al-Bederi O. N. H., Al-Sammarrae K. W. (2013). J. Assoc. Arab Univ. Basic Appl. Sci..

[cit68] Satpathy S., Patra A., Ahirwar B., Delwar H. M. (2018). Artif. Cells, Nanomed., Biotechnol..

[cit69] Yuan Y. G., Zhang S., Hwang J. Y., Kong I. K. (2018). Oxid. Med. Cell. Longevity.

[cit70] Haque S., Norbert C. C., Acharyya R., Mukherjee S., Kathirvel M., Patra C. R. (2021). Cancers.

[cit71] Nakkala J. R., Mata R., Raja K., Khub Chandra V., Sadras S. R. (2018). Mater. Sci. Eng., C.

[cit72] Tajkarimi M. M., Ibrahim G. A., Cliver D. O. (2010). Food Control.

[cit73] Catauro M., Raucci M. G., De Gaetano F. D., Marotta A. (2004). J. Mater. Sci.: Mater. Med..

[cit74] Crabtree J. H., Burchette R. J., Siddiqi R. A., Huen I. T., Hadnott L. L. (2003). Peritoneal Dial. Int..

[cit75] Zhang L., Ya J. C., Yip H. Y., Li Q., Kwong K. W., Xu A., Wong P. K. (2003). Langmuir.

[cit76] Pal S., Tak Y. K., Song J. M. (2007). Appl. Environ. Microbiol..

[cit77] Xia Z. K., Ma Q. H., Li S. Y., Zhang D. Q., Cong L., Tian Y. L., Yang R. Y. (2016). J. Microbiol., Immunol. Infect..

[cit78] Kim J. S., Kuk E., Yu K. N., Kim J. H., Park S. J., Lee H. J., Kim S. H., Park Y. K., Park Y. H., Hwang G. Y., Kim Y. K., Lee Y. S., Jeong D. H., Cho M. H. (2007). Nanomedicine.

[cit79] Kim K. J., Sung W. S., Suh B. K., Moon S. K., Choi J. S., Kim J. G., Lee D. G. (2009). BioMetals.

[cit80] Martinez-Castanon G. A., Nino-Martinez N., Martinez-Gutierrez F., Martinez-Mendoza J. R., Ruiz F. (2008). J. Nanopart. Res..

[cit81] Priya Velammal S., Devi T. A., Amaladhas T. P. (2016). J. Nanostruct. Chem..

[cit82] Granica S., Krupa K., Klebewska A., Kiss K. A. (2013). J. Pharm. Biomed. Anal..

[cit83] Senda J., Zieba J. (1972). Diss. Pharm. Pharmacol..

[cit84] Billa A. R., Palme E., Catalano S., Pistelli L., Morelli I. (1993). Fitoterapia.

[cit85] Billa A. R., Palme E., Catalano S., Pistelli L., Morelli I. A. (1993). Phytochemistry.

[cit86] Feng X. L., He Y. B., Liang Y. Z., Wang Y. I., Huang L. F., Xie J. W. (2013). J. Anal. Methods Chem..

[cit87] Elemike E. E., Fayemi O. E., Ekennia A. C., Onwudiwe D. C., Ebenso E. E. (2017). Molecules.

[cit88] Khorrami S., Zarrabi A., Khaleghi M., Danaei M., Mozafari M. R. (2018). Int. J. Nanomed..

