# Supplementary material for: Biosynthesis and characterization of silver nanoparticles synthesized using extracts of Agrimonia eupatoria L. and in vitro and in vivo studies of potential medicinal applications
Source: RSC Adv. 2024 Feb 5;14(7):4591–606. doi: 10.1039/d3ra07819a (PMC10839552; doi:10.1039/d3ra07819a)
Supplement: RA-014-D3RA07819A-s001 [file RA-014-D3RA07819A-s001.pdf]

Electronic Supplementary Material (ESI) for RSC Advances.
This journal is © The Royal Society of Chemistry 2024

Supplementary material

Biosynthesis and characterization of silver nanoparticles synthesized using extracts of *Agrimonia eupatoria* L. and *in vitro* and *in vivo* studies of potential medicinal applications

Katarina Marković[a], Ana Kesić[a]*, Mirjana Novaković[b], Mirjana Grujović[a], Dušica Simijonović[a], Edina H. Avdović[a], Sanja Matić[a], Milica Paunović[c], Milena Milutinović[c], Danijela Nikodijević[c], Olgica Stefanović[c] and Zoran Marković[a]

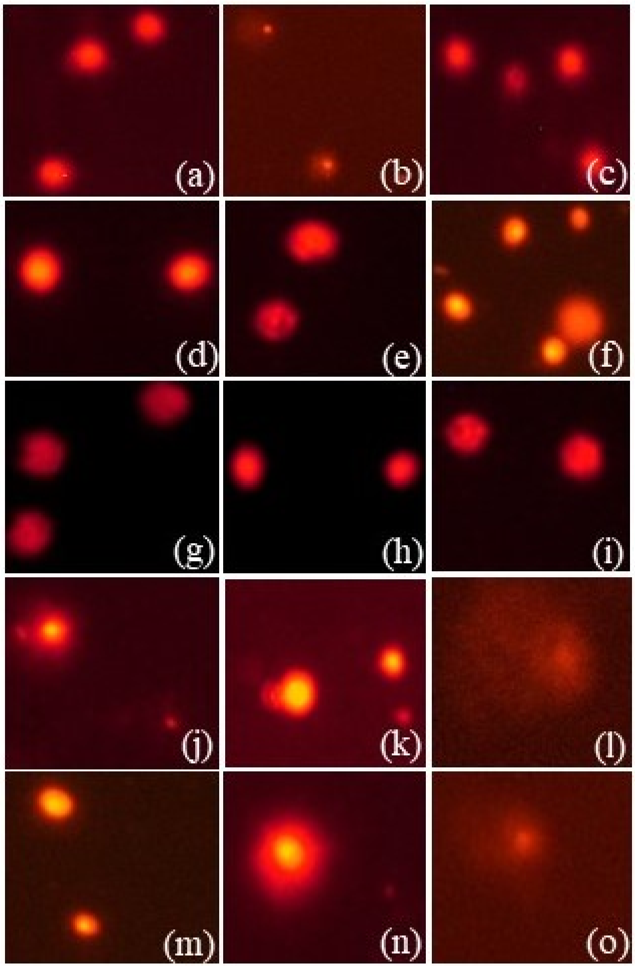

Figure S1. Representative alkaline comet assay images showing the DNA damage in the third instar larvae of *D. melanogaster* treated with different concentrations of the *Agrimonia eupatoria* water and acetone extract. Negative control (a), EMS (b), silver nitrate (c), water extract of *Agrimonia eupatoria* 0.5 mg/mL (d), water extract of *Agrimonia eupatoria* 1 mg/mL (e), water extract of *Agrimonia eupatoria* 2 mg/mL (f), acetone extract of *Agrimonia eupatoria* 0.5 mg/mL (g), acetone extract of *Agrimonia eupatoria* 1 mg/mL (h), acetone extract of *Agrimonia eupatoria* 2 mg/mL (i), water extract of *Agrimonia eupatoria* 0.5 mg/mL + 1 mM EMS (j), water extract of *Agrimonia eupatoria* 1 mg/mL + 1 mM EMS (k), water extract of *Agrimonia eupatoria* 2 mg/mL + 1 mM EMS (l), acetone extract of *Agrimonia eupatoria* 0.5 mg/mL + 1 mM EMS (m), acetone extract of *Agrimonia eupatoria* 1 mg/mL + 1 mM EMS (n) and acetone extract of *Agrimonia eupatoria* 2 mg/mL+ 1 mM EMS (o).

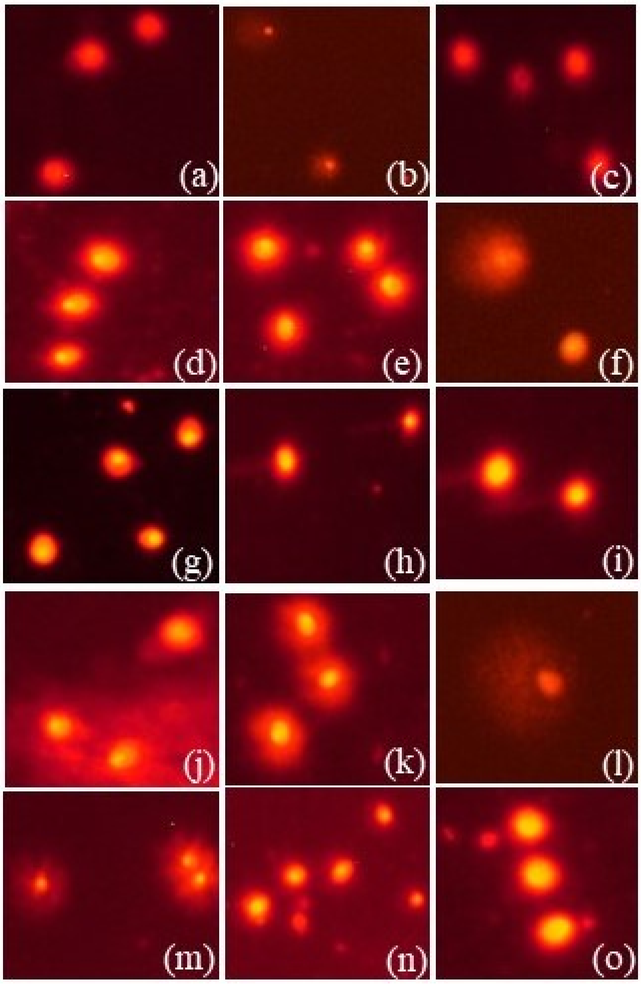

Figure S2. Representative alkaline comet assay images showing the DNA damage in the third instar larvae of *D. melanogaster* treated with different concentrations of the silver nanoparticles biosynthesized from water and acetone extracts of *Agrimonia eupatoria*. Negative control (a), EMS (b), silver nitrate (c), AgNPs-H_2O 0.5 mg/mL (d), AgNPs-H_2O 1 mg/mL (e), AgNPs-H_2O 2 mg/mL (f), AgNPs-acetone 0.5 mg/mL (g), AgNPs-acetone 1 mg/mL (h), AgNPs-acetone 2 mg/mL (i), AgNPs-H_2O 0.5 mg/mL + 1 mM EMS (j), AgNPs-H_2O 1 mg/mL + 1 mM EMS (k), AgNPs-H_2O 2 mg/mL + 1 mM EMS (l), AgNPs-acetone 0.5 mg/mL + 1 mM EMS (m), AgNPs-acetone 1 mg/mL + 1 mM EMS (n) and AgNPs-acetone 2 mg/mL+ 1 mM EMS (o).

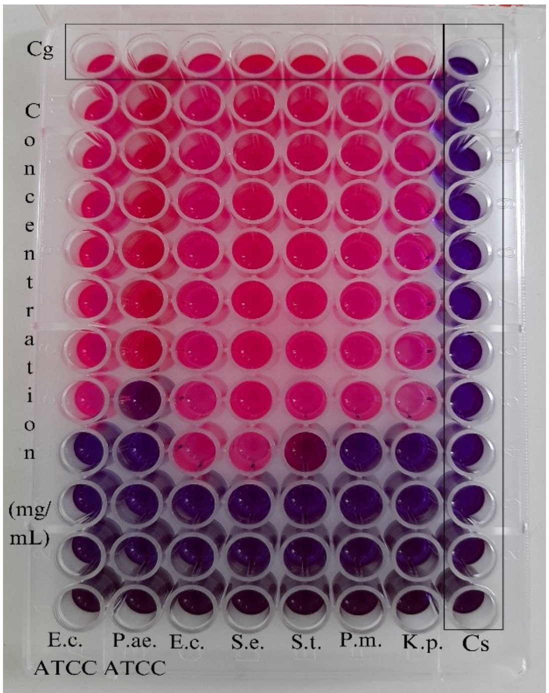

Figure S3. Effect of AgNPs – acetone on Gram negative bacteria (MIC values)
E.c. ATCC – *Escherichia coli* ATCC 25922; P.ae ATCC – *Pseudomonas aeruginosa* ATCC 27853; E.c. – *Escherichia coli*; S.e. – *Salmonella enterica*; S.t. – *Salmonella typhimurium*; P.m. – *Proteus mirabilis*; K.p. – *Klebsiella pneumoniae*; Cs – Sterility control; Cg – Growth control; Concentration range 5 – 0.0098 mg/ml

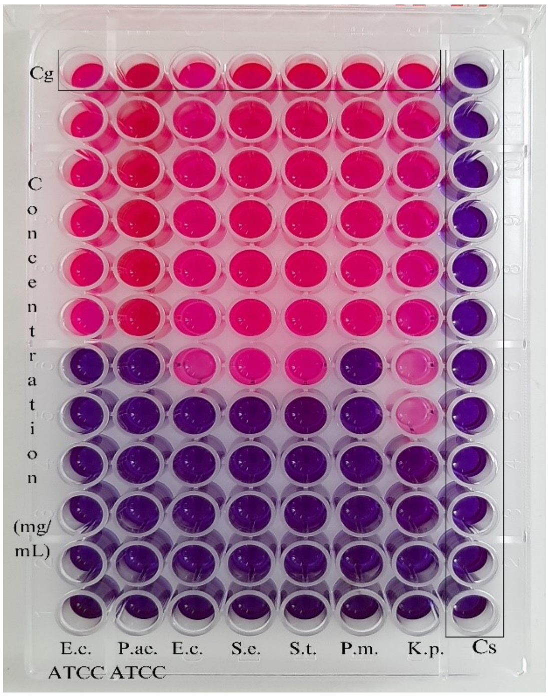

Figure S4. Effect of AgNPs – H_2O on Gram negative bacteria (MIC values)
E.c. ATCC – *Escherichia coli* ATCC 25922; P.ae ATCC – *Pseudomonas aeruginosa* ATCC 27853; E.c. – *Escherichia coli*; S.e. – *Salmonella enterica*; S.t. – *Salmonella typhimurium*; P.m. – *Proteus mirabilis*; K.p. – *Klebsiella pneumoniae*; Cs – Sterility control; Cg – Growth control; Concentration range 5 – 0.0098 mg/ml

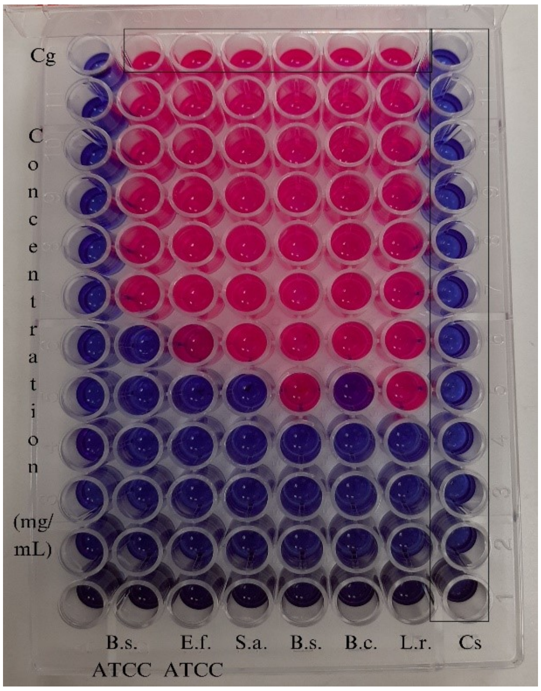

Figure S5. Effect of AgNPs – acetone on Gram positive bacteria (MIC values)
B.s ATCC – *Bacillus subtilis* ATCC 6633; E.f. ATCC – *Enterococcus faecalis* ATCC 29212; S.a. – *Staphylococcus aureus*; B.s. – *Bacillus subtilis*; B.c. – *Bacillus cereus*; L.r. – *Lactobacillus rhamnosus*; Cs – Sterility control; Cg – Growth control; Concentration range 5 – 0.0098 mg/ml

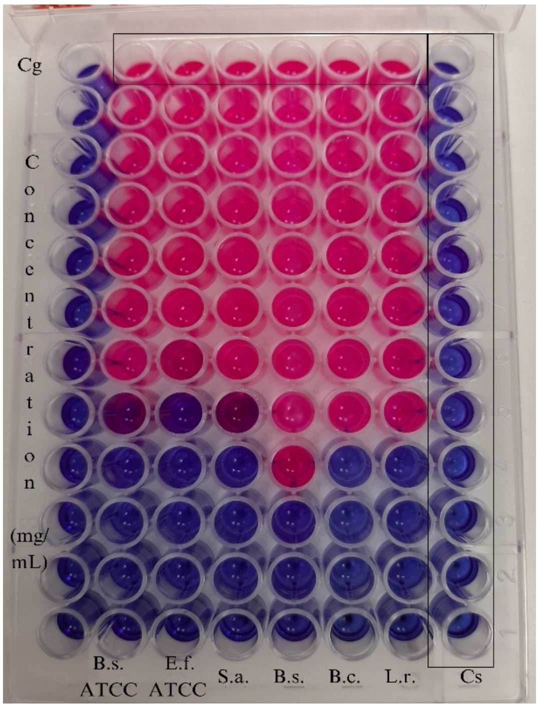

Figure S6. Effect of AgNPs – H_2O on Gram positive bacteria (MIC values)
B.s ATCC – *Bacillus subtilis* ATCC 6633; E.f. ATCC – *Enterococcus faecalis* ATCC 29212; S.a. – *Staphylococcus aureus*; B.s. – *Bacillus subtilis*; B.c. – *Bacillus cereus*; L.r. – *Lactobacillus rhamnosus*; Cs – Sterility control; Cg – Growth control; Concentration range 5 – 0.0098 mg/ml

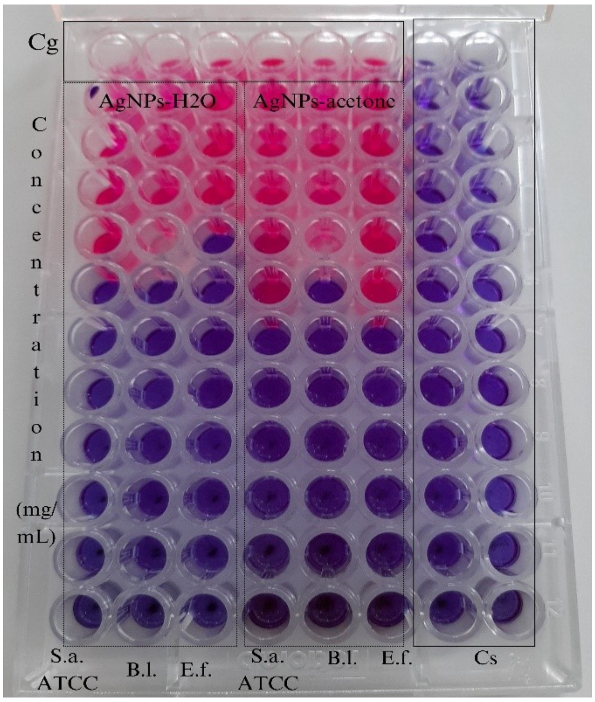

Figure S7. Effect of AgNPs – H_2O and AgNPs – acetone on Gram positive bacteria (MIC values)
S.a. ATCC – *Staphylococcus aureus* ATCC 25923; B.f. – *Bifidobacterium animalis* subsp. *lactis*; E.f. *Enterococcus faecalis*; Cs – Sterility control; Cg – Growth control; Concentration range 5 – 0.0098 mg/ml

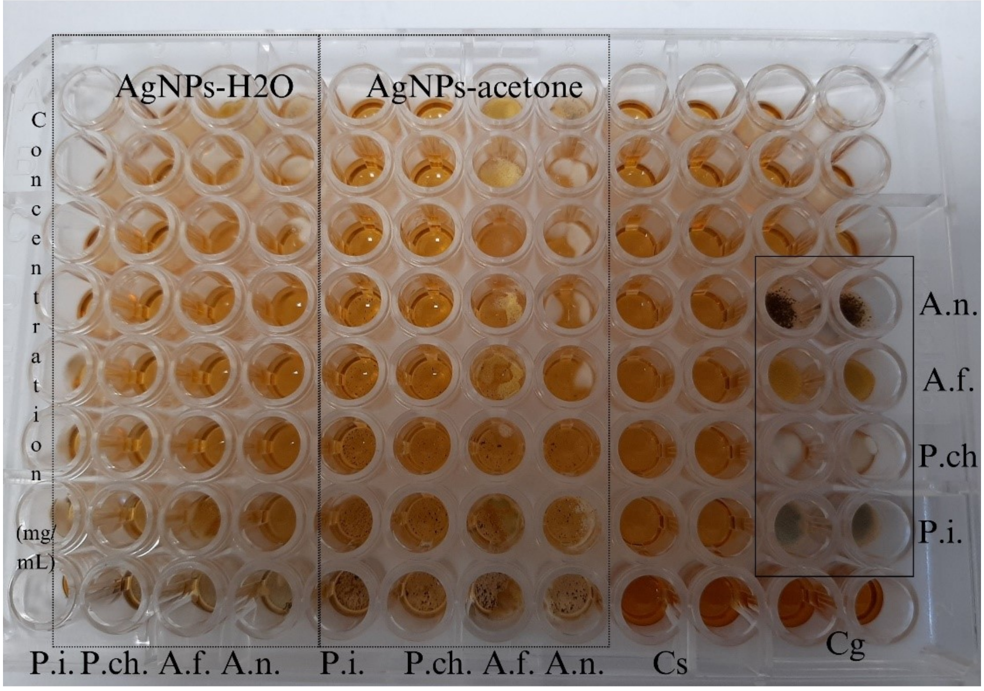

Figure S8. Effect of AgNPs – H_2O and AgNPs – acetone on filamentouse fungi (MIC values)
P.i. – *Penicillium italicum*; P.ch. – *Penicillium chrisogenum*; A.f. – *Aspergillus flavus*; A.n. – *Aspergillus niger*; Cs – Sterility control; Cg – Growth control; Concentration range 5 – 0.078 mg/ml

List of Supplementary figures

Figure S1. Representative alkaline comet assay images showing the DNA damage in the third instar larvae of *D. melanogaster* treated with different concentrations of the *Agrimonia eupatoria* water and acetone extract.
Negative control (a), EMS (b), silver nitrate (c), water extract of *Agrimonia eupatoria* 0.5 mg/mL (d), water extract of *Agrimonia eupatoria* 1 mg/mL (e), water extract of *Agrimonia eupatoria* 2 mg/mL (f), acetone extract of *Agrimonia eupatoria* 0.5 mg/mL (g), acetone extract of *Agrimonia eupatoria* 1 mg/mL (h), acetone extract of *Agrimonia eupatoria* 2 mg/mL (i), water extract of *Agrimonia eupatoria* 0.5 mg/mL + 1 mM EMS (j), water extract of *Agrimonia eupatoria* 1 mg/mL + 1 mM EMS (k), water extract of *Agrimonia eupatoria* 2 mg/mL + 1 mM EMS (l), acetone extract of *Agrimonia eupatoria* 0.5 mg/mL + 1 mM EMS (m), acetone extract of *Agrimonia eupatoria* 1 mg/mL + 1 mM EMS (n) and acetone extract of *Agrimonia eupatoria* 2 mg/mL+ 1 mM EMS (o).

Figure S2. Representative alkaline comet assay images showing the DNA damage in the third instar larvae of *D. melanogaster* treated with different concentrations of the silver nanoparticles biosynthesized from water and acetone extracts of *Agrimonia eupatoria*.
Negative control (a), EMS (b), silver nitrate (c), AgNPs-H_2O 0.5 mg/mL (d), AgNPs-H_2O 1 mg/mL (e), AgNPs-H_2O 2 mg/mL (f), AgNPs-acetone 0.5 mg/mL (g), AgNPs-acetone 1 mg/mL (h), AgNPs-acetone 2 mg/mL (i), AgNPs-H_2O 0.5 mg/mL + 1 mM EMS (j), AgNPs-H_2O 1 mg/mL + 1 mM EMS (k), AgNPs-H_2O 2 mg/mL + 1 mM EMS (l), AgNPs-acetone 0.5 mg/mL + 1 mM EMS (m), AgNPs-acetone 1 mg/mL + 1 mM EMS (n) and AgNPs-acetone 2 mg/mL+ 1 mM EMS (o).

Figure S3. Effect of AgNPs – acetone on Gram negative bacteria (MIC values)
E.c. ATCC – *Escherichia coli* ATCC 25922; P.ae ATCC – *Pseudomonas aeruginosa* ATCC 27853; E.c. – *Escherichia coli*; S.e. – *Salmonella enterica*; S.t. – *Salmonella typhimurium*; P.m. – *Proteus mirabilis*; K.p. – *Klebsiella pneumoniae*; Cs – Sterility control; Cg – Growth control; Concentration range 5 – 0.0098 mg/ml

Figure S4. Effect of AgNPs – H_2O on Gram negative bacteria (MIC values)
E.c. ATCC – *Escherichia coli* ATCC 25922; P.ae ATCC – *Pseudomonas aeruginosa* ATCC 27853; E.c. – *Escherichia coli*; S.e. – *Salmonella enterica*; S.t. – *Salmonella typhimurium*; P.m. – *Proteus mirabilis*; K.p. – *Klebsiella pneumoniae*; Cs – Sterility control; Cg – Growth control; Concentration range 5 – 0.0098 mg/ml

Figure S5. Effect of AgNPs – acetone on Gram positive bacteria (MIC values)
B.s ATCC – *Bacillus subtilis* ATCC 6633; E.f. ATCC – *Enterococcus faecalis* ATCC 29212; S.a. – *Staphylococcus aureus*; B.s. – *Bacillus subtilis*; B.c. – *Bacillus cereus*; E.f. – *Enterococcus faecalis*; L.r. – *Lactobacillus rhamnosus*; Cs – Sterility control; Cg – Growth control; Concentration range 5 – 0.0098 mg/ml

Figure S6. Effect of AgNPs – H_2O on Gram positive bacteria (MIC values)
B.s ATCC – *Bacillus subtilis* ATCC 6633; E.f. ATCC – *Enterococcus faecalis* ATCC 29212; S.a. – *Staphylococcus aureus*; B.s. – *Bacillus subtilis*; B.c. – *Bacillus cereus*; E.f. – *Enterococcus faecalis*; L.r. – *Lactobacillus rhamnosus*; Cs – Sterility control; Cg – Growth control; Concentration range 5 – 0.0098 mg/ml

Figure S7. Effect of AgNPs – H_2O and AgNPs – acetone on Gram positive bacteria (MIC values)
S.a. ATCC – *Staphylococcus aureus* ATCC 25923; E.f. *Enterococcus faecalis*; B.f. – *Bifidobacterium animalis* subsp. *lactis*; Cs – Sterility control; Cg – Growth control; Concentration range 5 – 0.0098 mg/ml

Figure S8. Effect of AgNPs – H_2O and AgNPs – acetone on filamentouse fungi (MIC values)
P.i. – *Penicillium italicum*; P.ch. – *Penicillium chrisogenum*; A.f. – *Aspergillus flavus*; A.n. – *Aspergillus niger*; Cs – Sterility control; Cg – Growth control; Concentration range 5 – 0.039 mg/ml
